# Cost-Utility Analysis of Venous Thromboembolism Prophylaxis Strategies for People Undergoing Elective Total Hip and Total Knee Replacement Surgeries in the English National Health Service

**DOI:** 10.3389/fphar.2018.01370

**Published:** 2018-11-27

**Authors:** Dalia M. Dawoud, David Wonderling, Jessica Glen, Sedina Lewis, Xavier L. Griffin, Beverley J. Hunt, Gerard Stansby, Michael Reed, Nigel Rossiter, Jagjot Kaur Chahal, Carlos Sharpin, Peter Barry

**Affiliations:** ^1^Clinical Pharmacy Department, Faculty of Pharmacy, Cairo University, Giza, Egypt; ^2^Clinical and Pharmaceutical Sciences Department, School of Life and Medical Sciences, University of Hertfordshire, Hatfield, United Kingdom; ^3^National Guideline Centre, Royal College of Physicians-London, London, United Kingdom; ^4^Nuffield Department of Orthopaedics, Rheumatology, and Musculoskeletal Sciences, University of Oxford, Oxford, United Kingdom; ^5^Guy's and St Thomas' NHS Foundation Trust, London, United Kingdom; ^6^Northern Vascular Unit, Freeman Hospital, Newcastle University and Newcastle Hospitals, Newcastle upon Tyne, United Kingdom; ^7^Northumbria Healthcare NHS Foundation Trust, North Shields, United Kingdom; ^8^Department of Trauma & Orthopaedic Surgery, Basingstoke & North Hampshire Hospital, Basingstoke, United Kingdom; ^9^Barts Health NHS Trust, London, United Kingdom; ^10^University Hospitals of Leicester NHS Trust, Leicester, United Kingdom

**Keywords:** venous thromboembolism (VTE) prophylaxis, pharmacoeconomics, cost utility analysis (CUA), total knee replacement (TKR), total hip replacement (THR), direct-acting oral anticoagulants, NICE guideline

## Abstract

**Background:** Major orthopedic surgery, such as elective total hip replacement (eTHR) and elective total knee replacement (eTKR), are associated with a higher risk of venous thromboembolism (VTE) than other surgical procedures. Little is known, however, about the cost-effectiveness of VTE prophylaxis strategies in people undergoing these procedures.

**Aim:** The aim of this work was to assess the cost-effectiveness of these strategies from the English National Health Service perspective to inform NICE guideline (NG89) recommendations.

**Materials and Methods:** Cost-utility analysis, using decision modeling, was undertaken to compare 15 VTE prophylaxis strategies for eTHR and 12 for eTKR, in addition to “no prophylaxis” strategy. The analysis complied with the NICE Reference Case. Structure and assumptions were agreed with the guideline committee. Incremental net monetary benefit (INMB) was calculated, vs. the model comparator (LMWH+ antiembolism stockings), at a threshold of £20,000/quality-adjusted life-year (QALY) gained. The model was run probabilistically. Deterministic sensitivity analyses (SAs) were undertaken to assess the robustness of the results.

**Results:** The most cost-effective strategies were LMWH for 10 days followed by aspirin for 28 days (INMB = £530 [95% CI: -£784 to £1,103], probability of being most cost-effective = 72%) for eTHR, and foot pump (INMB = £353 [95% CI: -£101 to £665]; probability of being most cost-effective = 18%) for eTKR. There was considerable uncertainty regarding the cost-effectiveness ranking in the eTKR analysis. The results were robust to change in all SAs.

**Conclusions:** For eTHR, LMWH (standard dose) for 10 days followed by aspirin for 28 days is the most cost-effective VTE prophylaxis strategy. For eTKR, the results are highly uncertain but foot pump appeared to be the most cost-effective strategy, followed closely by aspirin (low dose). Future research should focus on assessing cost-effectiveness of VTE prophylaxis in the eTKR population.

## Introduction

Hospital-acquired venous thromboembolism (VTE), also referred to as hospital-acquired thrombosis (HAT), represents a major patient safety concern (Hauck et al., 2017). The Department of Health, in England, defines it as “any episode of VTE arising in the 90 days following admission to hospital” (Arya et al., 2010). VTE can occur in the deep veins of either the legs or pelvis [a deep vein thrombosis (DVT)] or can present as a pulmonary embolism (PE) which can be fatal (Hunt, 2016). Treatment of non-fatal HAT and related long-term morbidities is associated with considerable cost (Barco et al., 2016). In the USA, the 5-year costs were predicted to be 1.5-fold higher for patients with HAT following major surgery ($55,956) than for hospitalized controls ($32,718; *P* < 0.001). Litigation costs and financial penalties on hospitals as a result of failure to prevent HAT have added to this huge cost impact (Cohoon et al., 2015; White et al., 2015).

The incidence of HAT is estimated to represent around 50–60% of all VTE seen globally (ISTH Steering Committee for World Thrombosis Day, 2014). It was the leading cause of disability-adjusted life-years in low- and middle-income countries, and the second most common cause in high-income countries (ISTH Steering Committee for World Thrombosis Day, 2014). The National VTE prevention program in England mandates that VTE risk assessment should be undertaken upon admission to hospital and thromboprophylaxis started soon after, in line with the recommendations of the National Institute for Health and Care Excellence (NICE; National Clinical Guideline Centre, 2010). This has shown positive outcomes in terms of reducing the incidence of HAT (Roberts et al., 2013). Thromboprophylaxis includes mechanical (such as anti-embolism stockings [AES], foot impulse and intermittent pneumatic compression devices [IPCD]) and pharmacological interventions (such as low molecular weight heparins [LMWHs], fondaparinux, direct-acting oral anticoagulants [DOACs] and low-dose aspirin) which can be used alone or in combination. According to the recommendations of published clinical guidelines, the need for and choice of a thromboprophylaxis strategy should be based on the population and the outcome of VTE risk assessment (Gould et al., 2012; National Institute for Health and Care Excellence, 2018).

Major orthopedic surgery, such as elective total hip replacement (eTHR) and elective total knee replacement (eTKR), are associated with a higher risk of VTE than other surgical populations (National Clinical Guideline Centre, 2010). However, the use of pharmacological agents for thromboprophylaxis, particularly the DOACs, should be balanced against the increase in risk of post-operative bleeding as a result of anticoagulation (Dahl et al., 2010). Additionally, the routine use of VTE prophylaxis in these populations has a considerable cost impact for the units delivering care (Board NE, 2017). According to the 14th report of the National Joint Registry (NJR) for 2017; there were 101,651 hip replacement operations and 108,713 knee replacement operations in England, Wales and Northern Ireland (Board NE, 2017). The large majority of these operations are elective primary lower limb total joint replacement procedures. The use and choice of VTE prophylaxis in these high-volume procedures should ideally represent value for money and justify the use of the scarce healthcare resources.

A literature review of published economic evaluations of VTE prophylaxis in eTHR and eTKR populations, completed during the development of NICE guideline NG 89 (National Institute for Health and Care Excellence, 2018), identified 32 [(Davies et al., 2000; Dahl and Pleil, 2003; Gordois et al., 2003; Lundkvist et al., 2003; Annemans et al., 2004; Dranitsaris et al., 2004, 2009; Haentjens et al., 2004; Reeves et al., 2004; Bjorvatn and Kristiansen, 2005; Bischof et al., 2006; National Colloborating Centre for Acute Care, 2007; National Institute for Health and Clinical Excellence, 2008, 2009, 2012; McCullagh et al., 2009, 2012; Stevenson et al., 2009; Wolowacz et al., 2009, 2010; Capri et al., 2010; Diamantopoulos et al., 2010; National Clinical Guideline Centre, 2010; Braidy et al., 2011; Riemsma et al., 2011; Ryttberg et al., 2011; McDonald et al., 2012; Migliaccio-Walle et al., 2012; Postma et al., 2012; Zindel et al., 2012; Hamidi et al., 2013; Revankar et al., 2013; Gomez-Outes et al., 2014; Wade et al., 2015; Sterne et al., 2016)] and 30 studies (Gordois et al., 2003; Lundkvist et al., 2003; Annemans et al., 2004; Dranitsaris et al., 2004, 2009; Haentjens et al., 2004; Reeves et al., 2004; Bjorvatn and Kristiansen, 2005; Bischof et al., 2006; National Colloborating Centre for Acute Care, 2007; National Institute for Health and Clinical Excellence, 2008, 2009, 2012; Stevenson et al., 2009; Wolowacz et al., 2009, 2010; Capri et al., 2010; Diamantopoulos et al., 2010; National Clinical Guideline Centre, 2010; Braidy et al., 2011; Riemsma et al., 2011; Ryttberg et al., 2011; McCullagh et al., 2012; McDonald et al., 2012; Migliaccio-Walle et al., 2012; Postma et al., 2012; Zindel et al., 2012; Hamidi et al., 2013; Revankar et al., 2013; Gomez-Outes et al., 2014; Wade et al., 2015; Sterne et al., 2016) respectively. The interventions compared and the most cost-effective prophylaxis strategy varied among these studies. Hence; this economic analysis was undertaken to assess the cost effectiveness of thromboprophylaxis strategies for eTHR and eTKR populations in English National Health Service (NHS) hospitals to inform the recommendations of the updated NICE guideline (NG89) (National Institute for Health and Care Excellence, 2018). This analysis takes into account the risks, benefits and costs of all the currently available thromboprophylaxis options in England and addresses the limitations of previously published models and economic analyses in this area.

## Materials and methods

A cost-utility analysis (CUA) was conducted from the perspective of the NHS and Personal Social Services (PSS), with quality-adjusted life-years (QALYs) and costs as the main outcome measures. The analysis complied with the NICE Reference Case, which is a set of methodological standards specified by NICE including using lifetime time horizon and applying discounting at a 3.5% discount rate for costs and outcomes accrued beyond the first year (National Institute for Health and Care Excellence, 2014). The reporting of the study follows the Consolidated Health Economic Evaluation Reporting Standards (CHEERS) statement (Husereau et al., 2013). No ethics approval was required for this study.

### Comparators

Interventions included in the model are outlined in Table 1, below, for eTHR and eTKR populations. These were decided in discussion with the clinical experts and patient members of the guideline committee to reflect currently used prophylaxis strategies in the UK. They were identified through a systematic review (SR) of published randomized controlled trials (RCTs) and were included in network meta-analyses (NMAs) of the main outcomes of interest. The detail of the SR and NMAs is reported in appendix M of the full NICE guideline (NG89) (National Institute for Health and Care Excellence, 2018).

**Table 1:** Interventions included in the model by population.

	**Strategy**	**Duration (a)**
**Elective total hip replacement (eTHR)**		
None	No prophylaxis	Not applicable
Mechanical only	AES (above-knee) AES (length unspecified)	7 days10 days
	IPCD (length unspecified)	8 days
	Foot pump	7 days
	Foot pump + AES	7 days
Pharmacological only	LMWH [standard dose (b); standard duration]	14 days
	LMWH [standard dose (b); extended duration]	33 days
	Dabigatran (c)	32 days
	Rivaroxaban (d)	35 days
	Apixaban (e)	32 days
	Aspirin (f; low dose, standard duration)	7 days
	LMWH [standard dose(b), standard duration] followed by aspirin (low dose, extended duration)	10 days (LMWH) followed by 28 days (aspirin)
Combination (Pharmacological + mechanical)	LMWH [standard dose (b); standard duration] + AES	14 days (LMWH) +10 days (AES) +
	LMWH [standard dose (b); extended duration] + AES	33 days (LMWH) + 10 days (AES)
	Fondaparinux (g) + AES	8 days (fondaparinux) 10 days (AES)
**Elective total knee replacement (eTKR)**		
None	No prophylaxis	Not applicable
Mechanical only	AES (length unspecified)	11 days
	IPCD (length unspecified)	6 days
	Foot pump	4 days
	Foot pump + AES	4 days+ 11 days
Pharmacological only	LMWH [standard dose (b); standard duration]	10 days
	LMWH [standard dose (b); extended duration]	30 days
	Dabigatran (h)	11 days
	Rivaroxaban (d)	13 days
	Apixaban (e)	12 days
	Aspirin (f; low dose, standard duration)	14 days
Combination (Pharmacological + mechanical)	LMWH [standard dose (b); standard duration] + AES	10 days
	Fondaparinux (g) + AES	11 days

*AES, anti-embolism stockings; IPCD, intermittent pneumatic compressions device; LMWH, low molecular weight heparin*.

*^a^ Average dose and duration based on the randomized controlled trials identified in the systematic review and network meta-analysis*.

*^b^ LMWH standard prophylactic dose included enoxaparin sodium 40 mg once daily, Dalteparin sodium 5,000 iu once daily and tinzaparin sodium 4,500 iu once daily*.

*^c^ Dabigatran dose for THR: For Adult 18–74 years, 110 mg, to be taken 1–4 h after surgery, followed by 220 mg once daily. For Adult 75 years and over: 75 mg, to be taken 1–4 h after surgery, followed by 150 mg once daily to be taken 12–24 h after initial dose*.

*^d^ Rivaroxaban dose: 10 mg once daily*.

*^e^ apixaban dose: 2.5 mg twice daily*.

*^f^ aspirin dose 75 – 150 mg once daily*.

*^g^ fondaparinux dose: 2.5 mg once daily*.

*^h^ dabigatran dose for TKR: For Adult 18–74 years: 110 mg, to be taken 1–4 h after surgery, followed by 150 mg once daily, to be taken 12–24 h after initial dose. For Adult 75 years and over: 75 mg, to be taken 1–4 h after surgery, followed by 150 mg once daily, to be taken 12–24 h after initial dose*.

The systematic review was very comprehensive, including all thromboprophylaxis strategies that were assessed in randomized controlled trials including those that do not represent current practice in the UK (e.g., LMWH in doses of 60 mg per day and aspirin in doses up to 300 mg daily). This was decided to increase the power of the NMAs. For the economic evaluation, however, only interventions that were largely in line with current practice in the UK and, thus, are considered relevant to decision making were included.

For strategies that included LMWH, only those that included prophylactic rather than treatment doses were included. The range of prophylactic LMWH doses was based on published guidance and expert input from the pharmacist member of the committee and was confirmed by the other committee members. The term “standard dose” was used to describe LMWH prophylactic doses. Additionally, the duration of use was differentiated into either standard (10–14 days) or extended (28–35 days). It was assumed that all LMWHs used are the originator rather than biosimilar options.

For strategies including aspirin, daily doses of up to 300 mg of aspirin were included in the NMA. For the economic evaluation, we modeled these as including low-dose aspirin (75–150 mg) administered for either standard (10 to 14 days) or extended duration (28–35 days). Complete details of the strategies compared in the model are available in Appendix P of NG89 and are summarized here in Table 1.

### Model description

A decision analytic model was developed and run separately for each of the two populations to reflect the difference in baseline characteristics, VTE and bleeding risks and prophylaxis duration. However, the structure of the model was the same for both populations.

The model consisted of a simple decision tree covering the acute phase from admission up to 90 days post-operatively, to cover the period included in the definition of hospital-acquired VTE, followed by a Markov model for the remaining model time horizon (lifetime in the base case). Markov models use disease states to represent all possible consequences of an intervention of interest.

**Figure 1:**
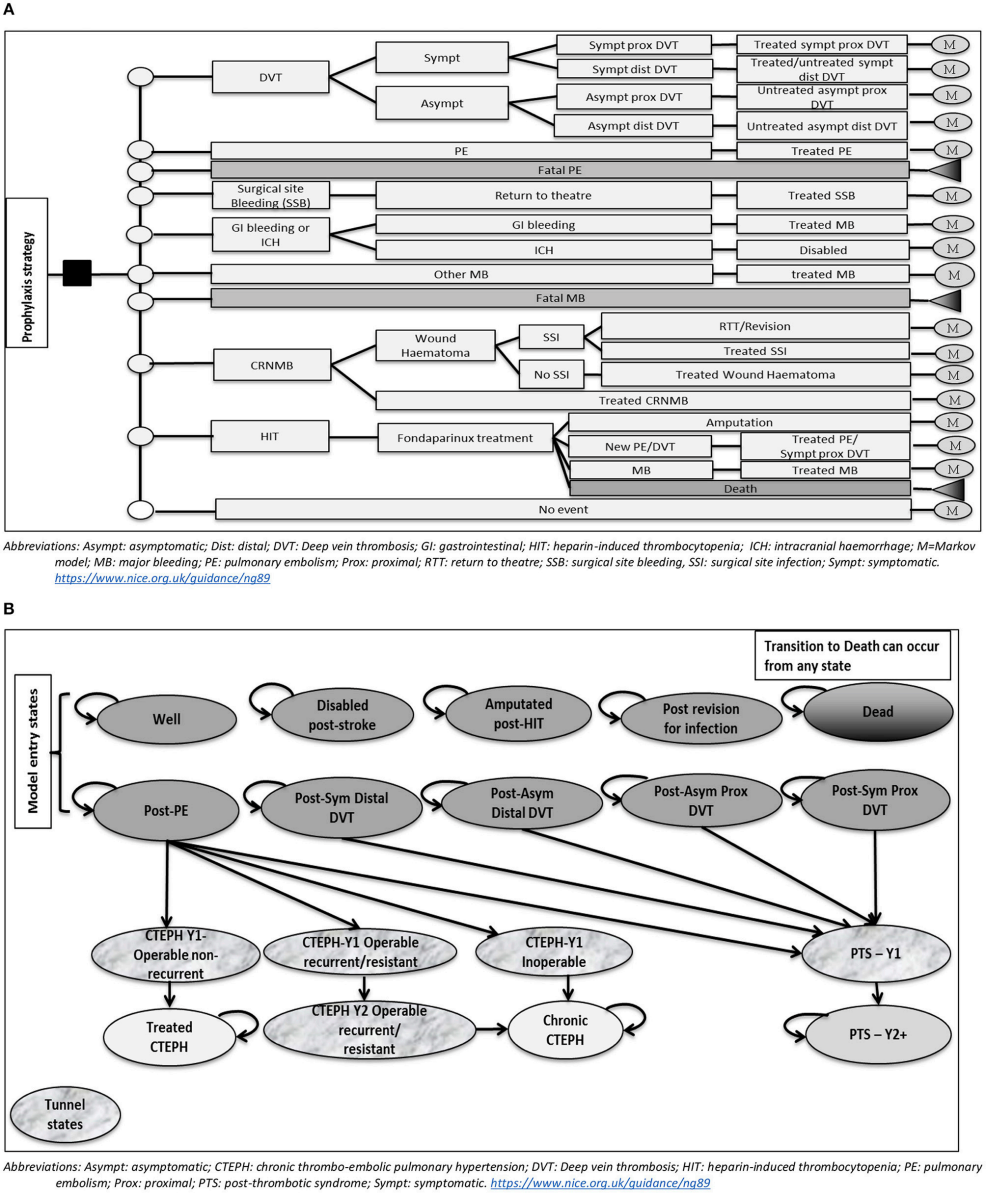
**(A)** Model structure up to 90 days post-operatively (Decision tree part). **(B)** Model structure after 90 days post-operatively (Markov model part).

The structure of the decision tree is presented in Figure 1A. It included the following events: DVT (symptomatic proximal, symptomatic distal, asymptomatic proximal, and asymptomatic distal), non-fatal PE, fatal PE, surgical site bleeding (SSB), non-surgical site bleeding (gastrointestinal (GI) bleeding, intracranial hemorrhage (ICH)/haemorrhagic stroke, other major bleeding), fatal major bleeding, clinically-relevant non-major bleeding (CRNMB), and heparin-induced thrombocytopaenia (HIT). Major bleeding (MB) events in the model could be at the surgical site; in which case it would result in return to theater, or at another site. MB occurring in the GI tract was assumed to require intervention in 13% of cases (National Clinical Guideline Centre, 2010). ICH/haemorrhagic stroke was assumed to lead to permanent disability.

Individuals who develop CRNMB were assumed to either be medically treated or develop a wound haematoma that could lead to a surgical site infection (SSI). SSIs could either be medically treated or require surgical intervention; which could be either a return to theater or a revision arthroplasty, in a ratio of 1:1. Individuals developing HIT were assumed to be treated with a therapeutic dose of fondaparinux. Outcomes included successful treatment, new thrombosis (assumed to be either symptomatic proximal DVT or PE in a ratio of 1:1), major bleeding or death.

The structure of the Markov cohort model is illustrated in Figure 1B. Individuals enter the model in one of the following states: Well, post-symptomatic proximal DVT, post-symptomatic distal DVT, post-asymptomatic proximal DVT, post-asymptomatic distal DVT, post-PE, amputated post-HIT, disabled post-stroke, post-revision for infection. In the first two years, individuals in a post-VTE state can develop post-thrombotic syndrome (PTS). Those in the post-PE state can also develop chronic thromboembolic pulmonary hypertension (CTEPH). The first year after the diagnosis of each of PTS or CTEPH is represented in the model by a distinct “tunnel” state, where individuals stay for one cycle only. Additionally, the second year after a recurrent/resistant CTEPH is represented by a “tunnel” state to account for the difference in costs from a chronic CTEPH state. Transitioning to death is allowed from any state in the model, to represent all-cause mortality.

A number of assumptions were made when developing the model. These were discussed in detail with and agreed by the guideline committee (see Panel 1).

Panel 1 **Main assumptions used in the model**

- Asymptomatic DVT is not diagnosed in practice and will not be treated or lead to extra costs or loss in quality of life in the short term.
- Only one symptomatic event is allowed in the model in the first 90 days; given that the treatment course for these events is 3 months long and once an event is diagnosed; the individual would receive treatments and would no longer be considered to be receiving primary prophylaxis.
- Those who develop symptomatic proximal DVT or PE will receive treatment. The treatment used was assumed to be either a direct oral anticoagulant (rivaroxaban or apixaban) or LMWH followed by vit-K antagonist (warfarin) in a ratio of 50% each. Dabigatran and edoxaban were excluded due to the initial 5-day dosing with LMWH following VTE diagnosis.
- It was assumed the treatment of VTE events is 100% effective, regardless of which VTE treatment regimen is used and no allowance for recurrence was made in the model. This was decided based on discussions with the committee where it was decided that the rate of recurrence after a provoked VTE is much lower compared to unprovoked VTE event. It was also felt that the prevention of a provoked event will not necessarily lead to prevention of recurrence which might be a result of a previous undiagnosed VTE event or an inherent susceptibility, including thrombophilia.
- The relative efficacy of the included comparators on the model outcomes was applied during the acute phase of the model, after which progression through the model was treatment-independent.

### Simulation cohort

The cohort characteristics for each population were based on data from the National Joint Registry (NJR) 13th annual report (Board NE, 2017), which were collected up to December 2015. These are presented in Table 2.

**Table 2:** Summary of base-case model inputs and their sources.

**Input**	**Data**	**Source**
**COHORT SETTINGS**		
Start age (years)	eTHR: 68.7 (SD = 11.32) eTKR: 69.3 (SD = 9.58)	National Joint Registry Annual Report 2016 (Board NE, 2017)
Male	eTHR: 40% eTKR: 44%	National Joint Registry Annual Report 2016 (Board NE, 2017)
BMI (kg/m^2^)	eTHR: 28.7 eTKR: 30.9	National Joint Registry Annual Report 2016 (Board NE, 2017)
**BASELINE RISKS–e THR**		
**DVT (symptomatic and asymptomatic)**	5.54%	Calculated based on (Jameson et al., 2011) and (Quinlan, 2007)
**Symptomatic DVT**	0.94%	(Jameson et al., 2011)
Proportion of symptomatic DVTs that are proximal	83.3%	(Revankar et al., 2013) based on data from ADVANCE trials
**Asymptomatic DVT**	4.6%	Calculated based on (Jameson et al., 2011) and (Quinlan, 2007)
Proportion of asymptomatic DVTs that are proximal	26.2%	(Revankar et al., 2013) based on data from ADVANCE trials
**Non-fatal PE**	0.68%	(Jameson et al., 2011)
Mortality from PE	17% (1/6)	Randomized controlled trials in our systematic review
Major bleeding at the surgical site	2.29%	Single-arm meta-analysis of the LMWH (standard dose, standard duration) randomized controlled trials in our systematic review
GI and cerebrospinal bleeding	0.72%	(Jameson et al., 2011)
Other major bleeding	0.2%	Single-arm meta-analysis of the LMWH (standard dose, standard duration) randomized controlled trials in our systematic review
Clinically-relevant non-major bleeding (CRNMB)	2.95%	Single-arm meta-analysis of the LMWH (standard dose, standard duration) randomized controlled trials in our systematic review
Wound haematoma as percentage of CRNMB	22.73% (5/22)	Calculated from the LMWH randomized controlled trials in our systematic review
Heparin-induced thrombocytopenia (HIT)	0.17%	Single-arm meta-analysis of the LMWH (standard dose, standard duration) randomized controlled trials in our systematic review
**BASELINE RISK - eTKR**		
**DVT (symptomatic and asymptomatic)**	14%	Calculated based on (Jameson et al., 2012) and (Quinlan, 2007)
**Symptomatic DVT**	0.63%	(Jameson et al., 2012)
Proportion of symptomatic DVTs that are proximal	20%	(Revankar et al., 2013) based on data from ADVANCE trials
**Asymptomatic DVT**	13.37%	Calculated based on (Jameson et al., 2012) and (Quinlan, 2007)
Proportion of asymptomatic DVTs that are proximal	8.8%	(Revankar et al., 2013) based on data from ADVANCE trials
**Non-fatal PE**	0.45%	(Jameson et al., 2012)
Mortality from PE	17%	assumed equal to eTHR as there were no events in the single trial of LMWH (standard dose, standard duration)+ AES
Major bleeding at the surgical site	0.64%	Single-arm meta-analysis of the LMWH (standard dose, standard duration) randomized controlled trials in our systematic review
GI and cerebrospinal bleeding	0.39%	(Jameson et al., 2012)
Other major bleeding	0.2%	Single-arm meta-analysis of the LMWH (standard dose, standard duration) randomized controlled trials in our systematic review
CRNMB	4.15%	Single-arm meta-analysis of the LMWH (standard dose, standard duration) randomized controlled trials in our systematic review
Wound haematoma as percentage of CRNMB	18.97% (11/58)	Calculated from the LMWH randomized controlled trials in our systematic review
HIT	0.92%	Single-arm meta-analysis of the LMWH (standard dose, standard duration) randomized controlled trials in our systematic review
**OTHER PARAMETERS**		
Proportion requiring return to theater after surgical site major bleeding	100%	Standard definition of major bleeding and expert opinion
Proportion requiring intervention after GI bleeding	13%	CG92 (National Clinical Guideline Centre, 2010)
Surgical site infection due to haematoma	25.77% (25/97)	Wang 2014 (Wang et al., 2014)
Probability of revision/return to theater due to infection	44% (11/25)	Wang 2014 (Wang et al., 2014)
**LONG TERM EVENTS**		
**2-year incidence of PTS after:**		
Symptomatic proximal DVT	40%	(Kahn et al., 2016) and committee Expert opinion
Symptomatic distal DVT	10%	(Heit et al., 2001; Botteman et al., 2002) and committee opinion
Asymptomatic proximal DVT	15%	(Wille-Jørgensen et al., 2005)
Asymptomatic distal DVT	3.75%	(Heit et al., 2001; Botteman et al., 2002)
Non-fatal PE	15%	Committee expert opinion
**Proportion of PTS that is severe**	23%	[(Wolowacz et al., 2009); average from 8 incidence studies]
**2-year incidence of CTEPH after non-fatal PE**	3.2% (95% CI: 1.5%−3.1%)	[(Ende-Verhaar et al., 2017); systematic review of incidence studies]
**CTEPH mortality**	20%	CG92 (National Clinical Guideline Centre, 2010)
**COSTS (£)**		
Symptomatic proximal DVT	eTHR: £457 eTKR: £457	NG89 (Venous thromboembolism in over 16s: reducing the risk of hospital-acquired deep vein thrombosis or pulmonary embolism), Appendix P (National Institute for Health and Care Excellence, 2018)
Symptomatic distal DVT	eTHR: £295 eTKR: £295	NG89 (Venous thromboembolism in over 16s: reducing the risk of hospital-acquired deep vein thrombosis or pulmonary embolism), Appendix P (National Institute for Health and Care Excellence, 2018)
Non-fatal PE	eTHR: £991 eTKR: £992	NG89 (Venous thromboembolism in over 16s: reducing the risk of hospital-acquired deep vein thrombosis or pulmonary embolism), Appendix P (National Institute for Health and Care Excellence, 2018)
Return to theater for surgical site bleeding	eTHR: £6,278 eTKR: £6,177	NHS Schedule for Reference Costs 2015–2016 [(Department of Health, 2016); unit cost for primary eTHR] NHS Schedule for Reference Costs 2015–2016 [(Department of Health, 2016); unit cost for primary eTKR]
GI bleeding with intervention	£2,409	NHS Schedule for Reference Costs 2015–2016 (Department of Health, 2016)
GI bleeding without intervention	£855	NHS Schedule for Reference Costs 2015–2016 (Department of Health, 2016)
**Haemorrhagic stroke**		
Acute event-admission	£4,354	Weighted Cost of non-elective long stay admission for stroke with CC score 0-3 to 16+. HRG codes AA35A to AA35F.NHS Schedule for Reference Costs 2015–2016 (Department of Health, 2016)
Acute event- other costs for the first 90 days	£3,255	Three month costs calculated based Weighted average cost of the cost of stroke dependent state and independent state in year 1 from CG144 [VTE management and thrombophilia testing] less the cost of the acute stroke admission (National Clinical Guideline Centre, 2012a) Costs inflated to 2015–2016.
Year 1–dependent state	£29,776	CG144 [VTE management and thrombophilia testing; (National Clinical Guideline Centre, 2012a)] Costs inflated to 2015–2016
Year 1–independent state	£4,971	CG144 [VTE management and thrombophilia testing; (National Clinical Guideline Centre, 2012a)] Costs inflated to 2015–2016
Year 2+ – dependent state	£15,108	CG144 [VTE management and thrombophilia testing; (National Clinical Guideline Centre, 2012a)] Costs inflated to 2015–2016
Year 2+ – independent state	£1,172	CG144 [VTE management and thrombophilia testing; (National Clinical Guideline Centre, 2012a)] Costs inflated to 2015–2016
**CRNMB (post-discharge)**	£242	Committee expert opinion (2 outpatient visits)
**Surgical site infection- medically treated**	£3,696	NHS Schedule for Reference Costs 2015–2016
**Revision surgery for infected joint**	eTHR: £19,514 eTKR: £19,203	Kallala 2015 and NHS Schedule for Reference Costs 2015–2016
**HIT**	£463	NHS Schedule for Reference Costs 2015–2016 (Department of Health, 2016)
**Amputation after HIT:**		
acute event	£10,300	CG 147 [Lower Limb Peripheral Arterial Disease; (National Clinical Guideline Centre, 2012b)] adjusted for inflation to 2015–2016 values
Year 1	£31,259	CG 147 [Lower Limb Peripheral Arterial Disease–(National Clinical Guideline Centre, 2012b)] adjusted for inflation to 2015–2016 values
Year 2+	£25,987	CG 147 [Lower Limb Peripheral Arterial Disease; (National Clinical Guideline Centre, 2012b)] adjusted for inflation to 2015–2016 values
**PTS**		
Mild/Moderate -Year 1	£841	Caprini 2003 (Caprini et al., 2003) converted to 2000 GBP OECD PPP conversion and inflated to 2015–2016 values
Mild/Moderate -Year 2+	£342	Caprini 2003 converted to 2000 GBP OECD PPP; (Organisation for Economic Co-operation Development (OECD), 2012) conversion factor and inflated to 2015–2016 values
Severe -Year 1	£3,824	Caprini 2003 converted to 2000 GBP OECD PPP conversion; (Organisation for Economic Co-operation Development (OECD), 2012) and inflated to 2015–2016 values
Severe -Year 2+	£1,680	Caprini 2003 converted to 2000 GBP OECD PPP conversion; (Organisation for Economic Co-operation Development (OECD), 2012) and inflated to 2015–2016 values
**CTEPH**		
Operable-Y1	£28,671	NG89 (Venous thromboembolism in over 16s: reducing the risk of hospital-acquired deep vein thrombosis or pulmonary embolism), Appendix P (National Institute for Health and Care Excellence, 2018)
Recurrent/Resistant- Year 1	£29,470	NG89 (Venous thromboembolism in over 16s: reducing the risk of hospital-acquired deep vein thrombosis or pulmonary embolism), Appendix P (National Institute for Health and Care Excellence, 2018)
Inoperable-Year 1	£9,677	NG89 (Venous thromboembolism in over 16s: reducing the risk of hospital-acquired deep vein thrombosis or pulmonary embolism), Appendix P (National Institute for Health and Care Excellence, 2018)
Recurrent/resistant- Year 2	£21,845	NG89 (Venous thromboembolism in over 16s: reducing the risk of hospital-acquired deep vein thrombosis or pulmonary embolism), Appendix P (National Institute for Health and Care Excellence, 2018)
Chronic-Year 2+	£13,967	NG89 (Venous thromboembolism in over 16s: reducing the risk of hospital-acquired deep vein thrombosis or pulmonary embolism), Appendix P (National Institute for Health and Care Excellence, 2018)
Treated CTEPH	£147	NG89 (Venous thromboembolism in over 16s: reducing the risk of hospital-acquired deep vein thrombosis or pulmonary embolism), Appendix P (National Institute for Health and Care Excellence, 2018)

*BMI, body mass index; CRNMB, clinically-relevant non-major bleeding; CTEPH, chronic thromboembolic pulmonary hypertension; DVT, deep vein thrombosis; eTHR, elective total hip replacement; eTKR, elective total knee replacement; GI, gastrointestinal; HIT, Heparin-induced thrombocytopenia; LMWH, low molecular weight heparin; PE, pulmonary embolism; PTS, post-thrombotic syndrome*.

### Baseline risks

Baseline risks of VTE and major bleeding were based on two large observational cohort studies that used the NJR data (Jameson et al., 2011, 2012). In both, data for England and Wales, linked to an administrative database of hospital admissions in the English National Health Service (Hospital Episode Statistics [HES] database), were analyzed. The two studies only reported symptomatic DVT events. Hence, we used the asymptomatic-to-symptomatic DVT as reported in Quinlan (2007), to calculate the number of asymptomatic DVT events that would have been observed in these populations (see Table 2).

### Relative treatment effects

#### DVT and PE

The risk ratios (RRs) for each prophylaxis strategy compared to LMWH (std/std) + AES were based on systematic reviews and network meta-analyses (NMAs) of the outcomes: all DVT (symptomatic and asymptomatic) and non-fatal PE. The detail of these systematic reviews and NMAs are reported in appendix M of the full guideline (NG89) (National Institute for Health and Care Excellence, 2018). The absolute risks for each prophylaxis strategy were calculated by multiplying the RRs obtained from the NMA by the baseline risk of each event. These are presented in Table 3. Where an intervention had data available for DVT or PE only, we assumed proportionality of effect on these events. This assumption has been tested in 11 one-way sensitivity analyses (SAs).

**Table 3:** Absolute risk (%) of model events.

**Strategy**	**DVT (symptomatic and asymptomatic) (95% CrI)**	**Non-fatal PE (95% CrI)**	**GI bleeding + ICH**	**SSB**	**Other major bleeding**	**CRNMB**
**eTHR**						
LMWH (std,std) + AES	5.54% (%5.39 to %5.70)	0.68% (%0.63 to %0.74)	0.72%	0.94%	0.30%	3.04%
LMWH (std,extd)+ AEs	4.03% (%0.53 to %14.34)	0.15% (%0.00 to %0.94)	0.77%	0.70%	0.23%	3.04%
Fondaparinux+ AES	3.25% (%0.46 to %11.43)	1.15% (%0.09 to %5.12)	1.40%	1.57%	0.51%	4.98%
Foot pump + AES	14.66% (%1.99 to %46.06)	1.48% (b)	0.34%	0.36%	0.12%	1.18%
IPCD	33.06% (%5.56 to %76.99)	5.28% (%0.15 to %31.35)	0.34%	0.36%	0.12%	1.18%
AES (above knee)	8.30% (%0.87 to %48.85)	10.21% (%0.00 to %88.30)	0.34%	0.36%	0.12%	1.18%
Foot pump	28.01% (%2.41 to %78.81)	21.94% (%0.11 to %98.05)	0.34%	0.36%	0.12%	1.18%
AES	12.05% (%4.35 to %25.55)	1.18% (%0.08 to %5.46)	0.34%	0.36%	0.12%	1.18%
LMWH (std,std)	20.30% (%3.41 to %56.46)	2.47% (%0.18 to %12.53)	0.72%	0.94%	0.30%	3.04%
LMWH (std,extd)	9.76% (%0.97 to %36.66)	0.45% (%0.00 to %3.19)	0.77%	0.70%	0.23%	3.04%
Aspirin (std duration)	26.26% (%1.56 to %80.91)	36.63% (%0.35 to %99.62)	0.79% (c)	1.03%	0.33%	3.29%
LMWH (std, std) + Aspirin (extd duration)	0.05%(a)	0.11% (%0.00 to %0.77)	0.80%	0.10%	0.03%	1.64%
Dabigatran	18.91% (%2.05 to %60.30)	3.56% (%0.13 to %20.41)	1.19%	1.34%	0.43%	3.48%
Apixaban	9.81% (%0.55 to %43.30)	2.01% (%0.05 to %12.24)	1.17%	1.16%	0.37%	2.75%
Rivaroxaban	4.00% (%0.27 to %18.33)	1.20% (%0.01 to %7.82)	0.95%	0.99%	0.32%	3.68%
No prophylaxis	40.42% (%9.59 to %81.09)	8.80% (%0.83 to %37.52)	0.34%	0.36%	0.12%	1.18%
**eTKR**						
LMWH (std,std) + AES	14.00% (%13.81 to %14.20)	0.45% (%0.41 to %0.49)	0.39%	0.94%	0.21%	4.89%
Fondaparinux+ AES	12.51% (%3.76 to %27.50)	0.36% (d)	4.20%	5.85%	1.34%	25.11%
Foot pump + AES	18.96% (%9.45 to %33.25)	0.58% (d)	0.36%	0.88%	0.19%	4.58%
IPCD	21.23% (%7.04 to %42.74)	1.92% (%0.00 to %18.60)	0.36%	0.88%	0.19%	4.58%
Foot pump	8.38% (%1.12 to %26.89)	0.20% (d)	0.36%	0.88%	0.19%	4.58%
AES	29.97% (%15.13 to %48.19)	2.48% (%0.007 to %20.33)	0.36%	0.88%	0.19%	4.58%
LMWH (std,std)	9.22% (%2.98 to %20.08)	1.94% (%0.00 to %19.44)	0.39%	0.94%	0.21%	4.89%
LMWH (std,extd)	7.83% (%1.80 to %20.51)	0.87% (%0.000 to %6.25)	0.43%	0.14%	0.03%	6.77%
Aspirin	15.28% (%3.64 to %37.46)	0.43% (d)	0.38% (e)	0.93%	0.21%	4.84%
Dabigatran	9.10% (%2.78 to %20.49)	5.06% (%0.00 to %60.15)	0.44%	0.95%	0.21%	5.46%
Apixaban	5.31% (%1.54 to %12.44)	4.35% (%0.000 to %49.77)	0.34%	0.69%	0.15%	3.78%
Rivaroxaban	4.32% (%1.17 to %10.42)	1.45% (%0.00 to %13.84)	0.64%	1.33%	0.29%	5.83%
No prophylaxis	34.21% (%13.98 to %58.93)	4.47% (%0.002 to %46.25)	0.42%	0.88%	0.19%	4.58%

*AES, anti-embolism stockings; CrI, credible interval; CRNMB, clinically-relevant non-major bleeding; DVT, deep vein thrombosis; eTHR, elective total hip replacement; eTKR, elective total knee replacement; extd, extended; GI, gastrointestinal; ICH, intracranial hemorrhage; IPCD, intermittent pneumatic compressions device; LMWH, low molecular weight heparin; PE, pulmonary embolism; SSB: surgical site bleeding; std, standard*.

*^a^ Not in DVT NMA. Point estimate calculated based on the assumption that the relative effectiveness for the PE outcome compared to LMWH (std,std) + AES will be the same for the DVT*.

*^b^ Not in PE NMA. Point estimate calculated based on the assumption that the relative effectiveness for the DVT outcome compared to LMWH (std,std)+ AES will be the same for the PE*.

*^c^ Source: Jameson 2011 (Hauck et al., 2017)*.

*^d^ Not in PE network. Point estimate calculated based on the assumption that the relative effectiveness for the DVT outcome compared to LMWH (std,std)+ AES will be the same for the PE*.

*^e^ Source: Jameson 2012 (Arya et al., 2010)*.

#### Bleeding events

The relative efficacy of the included interventions compared to LMWH (std,std)+AES was calculated from systematic review and NMA of non-fatal MB reported in appendix M of the full NICE guideline (NG89; National Institute for Health and Care Excellence, 2018). In the model, we use these ORs and the baseline risk on LMWH (std,std)+AES to calculate the absolute risk of each MB event in the model. These ORs were also used to calculate the absolute risk of CRNMB when an intervention did not have trial data for this outcome. Wound haematoma and subsequent SSI were modeled as consequences of CRNMB, based on epidemiological data.

In the MB NMA, we assumed that the major bleeding rate for mechanical only strategies is the same as for the no prophylaxis strategy and these were treated as one intervention (National Institute for Health and Care Excellence, 2018). This was considered reasonable on biological grounds. No other complications from mechanical prophylaxis were included in the model. However, given the established evidence that some patients find stockings uncomfortable (Wade et al., 2015), causing patients to wear them incorrectly (especially thigh-length stockings), we included the cost of nurse time required for checking that mechanical prophylaxis is fitted correctly. The absolute risks of the bleeding events for each prophylaxis strategy are presented in Table 3.

### Utilities

A SR was conducted to identify utility inputs to use in the model. Additionally, we examined the sources used in the published economic evaluations and existing NICE Technology Appraisals (TAs) (National Institute for Health and Clinical Excellence, 2009, 2012; National Clinical Guideline Centre, 2010; National Institute for Health and Care Excellence, 2015).

For baseline utility values, we used EQ-5D-3L index values reported in the UK 2014-2015 PROMS programme (NHS Digital, 2016). The PROMS programme collects EQ-5D-3L data pre- and 6 months post-operatively for eTHR and eTKR patients. The post-operative EQ-5D-3L index values reported in the PROMS data represent the utility at 6–12 months. We assumed that this value would be reached at the mid-point (9 months). We also assumed a linear increase from the pre-operative utility over the 6 months (180 days) to calculate the utility score at 90 days (the point of entry to the Markov model).

Event-specific (Dis) utilities were applied as event-based after which the individual's QoL recovers and continues on the post-operative linear improvement trajectory to achieve the utility value at 90-days post-operatively. For the purpose of calculating QALYs, it was assumed that DVT and any adverse events take place on day 7 while PE events take place on day 21. This was based on committee estimates. Data from Warwick 2007 were used in sensitivity analysis (Warwick et al., 2007). All (dis)utility values are listed in Tables 4, 5.

**Table 4:** Base case (dis-)utility values for events up to 90 days.

	**Mean (dis-)utility**	**SE(a)**	**Source**
No event (baseline utility at 90 days)	THR: 0.579 (BLU-THR)	0.057	PROMS 2014–2015 (Hunt, 2016)
	TKR: 0.582 (BLU-TKR)	0.058	PROMS 2014–2015 (Hunt, 2016)
Asymptomatic DVT- Distal	THR: 0.579 (BLU-THR)	0.057	PROMS 2014–2015 (Hunt, 2016)
Asymptomatic DVT- Proximal	TKR: 0.582 (BLU-TKR)	0.058	PROMS 2014–2015 (Hunt, 2016)
Symptomatic DVT- Proximal	−14%		Cohen 2014 (Barco et al., 2016)
Symptomatic DVT- Distal (requiring treatment)	−14%		Assumption: equal to the disutility for symptomatic DVT-proximal
Symptomatic DVT- Distal (not requiring treatment)	−7%		Assumption: equal to the 50% of the disutility for symptomatic DVT-proximal
Non-fatal PE	−19%		Cohen 2014 (Barco et al., 2016)
Warfarin treated DVT or PE	−0.012		Marchetti 2001 (White et al., 2015) and Edoxaban TA354 (Cohoon et al., 2015) company submission
Major bleeding (surgical site, GI with or without intervention, other)	−32%		Locadia 2004 (ISTH Steering Committee for World Thrombosis Day, 2014)
ICH/acute stroke	−65%		Locadia 2004 (ISTH Steering Committee for World Thrombosis Day, 2014)
Pre- aseptic revision surgery	THR: 0.399	0.039	PROMS 2014–2015 (Hunt, 2016)
	TKR: 0.329	0.033	PROMS 2014–2015 (Hunt, 2016)
Post-aseptic revision surgery	THR: 0.538	0.054	PROMS 2014–2015 (Hunt, 2016)
	TKR: 0.459	0.046	PROMS 2014–2015 (Hunt, 2016)
Post-reoperation for surgical site MB	THR: 0.538	0.054	Assumed equal to post-aseptic revision
	TKR: 0.459	0.046	Assumed equal to post-aseptic revision
CRNMB (including wound haematoma)	−0.03		Sullivan 2011 (National Clinical Guideline Centre, 2010)
Surgical site infection	−66%		Baker 2013 (Roberts et al., 2013) for TKR, assumed the same for THR
Post-infected revision/return to theater for surgical site infection	−30%		Baker 2013 (Roberts et al., 2013) for TKR, assumed the same for THR
HIT	−0.0712		Gould 1999 (Gould et al., 2012)
Post-HIT amputation	−0.28		Beaudet 2014, T1D GL (Dahl et al., 2010)
Post-HIT thrombosis	−16.5%		Assumed average of PE and symptomatic proximal DVT dis-utilities
Post-HIT MB	−32%		Assumed equal to Major bleeding (surgical site, GI with or without intervention, other)

*BLU, baseline utility; CRNMB, clinically-relevant non-major bleeding; GI, gastrointestinal; HIT, heparin-induced thrombocytopaenia; ICH, intracranial hemorrhage; MB, major bleeding; PE, pulmonary embolism; SE, standard error; THR, total hip replacement; TKR, total knee replacement*.

*^a^ Where not reported; SE was calculated as 10% of the mean*.

**Table 5:** Base case (dis-)utility values for the Markov model health states (more than 90 days after surgery).

	**Mean (dis-)utility**	**SE(a)**	**Source**	**duration**
Post stroke (disabled)	−10%		Lunde 2013 (Board NE, 2017) 345 Stroke patients in Norway who had ischaemic/haemorrhagic or TIA	lifetime
Mild to Moderate PTS	−0.02		Lenert 1997 (Annemans et al., 2004)	lifetime
Severe PTS	−0.07		Lenert 1997 (Annemans et al., 2004)	lifetime
CTEPH-Year 1	−26%		Meads 2008 (Bischof et al., 2006)	Operable or inoperable (3 months) Recurrent/resistant (12 months)
CTEPH - Year 2- recurrent resistantChronic CTEPH	22%		Meads 2008 (Bischof et al., 2006)	Utility improvement after medical treatment applied to CTEPH-Year 1 utility value Chronic CTEPH utility applied lifetime
Post-HIT amputation	−0.28		Beaudet 2014 (Dahl et al., 2010), T1D GL (Bjorvatn and Kristiansen, 2005)	Lifetime

*HIT, heparin-induced thrombocytopaenia; SE, standard error; T1D, Type 1 diabetes*.

*^a^ Where not reported; SE was calculated as 10% of the mean*.

### Resource use and costs

Only direct medical costs calculated from the perspective of the English NHS were included in the analysis. These included interventions' costs and costs of model events occurring in the acute and long-term phase of the model.

#### Intervention costs

Prophylaxis costs were calculated based on the dose and duration used the in RCTs of each of its components (pharmacological and/or mechanical). A sensitivity analysis using the licensed doses and durations of pharmacological prophylaxis instead was also conducted. The administration and monitoring costs were included, where required. Intervention costs are presented in Table 6.

**Table 6:** Total costs of each prophylaxis strategy in the eTHR and eTKR models.

**Population and strategy**	**Total costs of pharmacological prophylaxis (I)**	**Total costs of mechanical prophylaxis (II)**	**Total intervention cost (I+II)**
**THR**			
LMWH (std,std) + AES	£138	£31	£169
LMWH (std,extd)+ AES	£387	£31	£419
Fondaparinux+ AES	£83	£31	£115
Foot pump + AES	£0	£91	£91
IPCD	£0	£42	£42
AES (above knee)	£0	£34	£34
Foot pump	£0	£59	£59
AES	£0	£31	£31
LMWH (std,std)	£138	£0	£138
LMWH (std,extd)	£387	£0	£387
Aspirin (std duration)	£0.2	£0	£0.2
LMWH (std, std) + Aspirin (extd duration)	£115	£0	£115
Dabigatran	£80	£0	£80
Apixaban	£59	£0	£59
Rivaroxaban	£74	£0	£74
No prophylaxis	£0	£0	£0
**TKR**			
LMWH (std,std) + AES	£111	£31	£142
Fondaparinux+ AES	£97	£31	£128
Foot pump + AES	£0	£91	£91
IPCD	£0	£42	£42
Foot pump	£0	£59	£59
AES	£0	£31	£31
LMWH (std,std)	£111	£0	£111
LMWH (std,extd)	£355	£0	£355
Aspirin	£0.5	£0	£0.5
Dabigatran	£34	£0	£34
Apixaban	£23	£0	£23
Rivaroxaban	£25	£0	£25
No prophylaxis	£0	£0	£0

*AES, anti-embolism stockings; eTKR, elective total knee replacement; eTHR, elective total hip replacement; extd, extended; IPCD*.

#### Event costs

We calculated the costs of diagnosing and treating the model events. Resources required were identified through discussions with the committee members and examining previously published models, NICE Technology Appraisals (TAs) and clinical guidelines. These resources were then identified and valued according to standard methods including micro-costing using bottom-up and top-down approaches, as required (National Institute for Health and Care Excellence, 2014). The resources considered were those of diagnostic tests, drug treatments, staff time, primary care, outpatient, and emergency department visits, ICU admissions, ambulance transfer and re-operation.

The costs of diagnosing, short and long term management of the following events were included: symptomatic DVT, PE, MB (gastrointestinal hemorrhage, surgical site bleeding, intracranial hemorrhage (stroke), bleeding at any other site), CRNMB, SSI, HIT, amputation as a result of HIT, CTEPH, severe and mild to moderate PTS and post-stroke disability.

National sources of unit costs in the UK were used including the Drug Tariff, British National Formulary (BNF), NHS Supply Chain Catalog and NHS Schedule for Reference Costs (Department of Health, 2015; NHS Business Services Authority, 2015a,b; Curtis and Burns, 2016; Joint Formulary Committee, 2016). The price year was 2016. Any costs from earlier years were adjusted for inflation using the Hospital and Community Health Services Pay and Prices Index (Curtis and Burns, 2016). Costs based on published literature from other countries were converted to 2016 GBP using Organization for Economic Co-operation and Development (OECD) purchasing power parity (PPP) calculator (Organisation for Economic Co-operation Development (OECD), 2012). Detailed calculations of the costs used in the analysis are reported in Appendix P of NG89 and are summarized here in Table 2 (National Institute for Health and Care Excellence, 2018).

### Sensitivity analyses (SAs)

A number of one-way deterministic sensitivity analyses were undertaken to assess uncertainty in the model. These were as follows: changing the cost-effectiveness threshold from £20,000 to £30,000 per QALY-gained (*SA1*), changing the discount rate from 3.5% to 1.5% (*SA2*), changing the source of the pharmacological prophylaxis duration from being based on the RCTs to their licensed durations (*SA3*), changing the cohort starting age to 40 years (*SA4*), changing the cohort body weight from the NJR cohort mean weight to a distribution of body weights calculated based on the NJR cohort BMI distribution and average height for a UK male (1.75 m) and female (1.62 m; *SA5*), increasing all intervention and event costs by 10% (*SA6*), decreasing all costs by 10% (*SA7*), changing the timing of VTE and MB events to be based on data from Warwick 2007 (*SA8*; Warwick et al., 2007), using alternative rates of recurrence for treated DVT and PE based on data from TA245 and TA354 manufacturer submissions (*SA9*; National Institute for Health and Clinical Excellence, 2012; National Institute for Health and Care Excellence, 2015), taking wastage into account when calculating the costs of pharmacological prophylaxis (*SA10*) and using alternative value for the risk of DVT when using LMWH (std,std) followed by aspirin for the eTHR population which is calculated using the odds ratio from Anderson 2013 for the outcome proximal DVT rather than using the estimate from the PE NMA results [*SA11*; (Anderson et al., 2013)].

### Analysis approach

The model was constructed in Microsoft Excel 2010 and was evaluated by cohort simulation. Time dependency was built in the long-term Markov part of the model by cross referencing the cohorts age as a respective risk factor for mortality. Baseline utility was also time dependent and was conditional on the number of years after entry to the model.

Patients start in cycle 0 in the health state corresponding to the end state of the decision tree part of the model. Patients moved to the dead health state at the end of each cycle as defined by the mortality transition probabilities from the life tables and CTEPH mortality.

PTS and CTEPH incidence rates were converted into transition probabilities for the respective cycle length (1 year in the base case) before inputting into the Markov model. These conversions were done using the following formulae:

$$\begin{array}{l} \begin{array}{cc} Selected\;rate\;\left(r\right) & =\;\frac{-\ln\left(1-P\right)}{t}\\ \text{Where}\\ P & =\text{probability of event over time }t\\ t & =\text{time over which probability occurs}\left(2\text{ years}\right)\\ Transition\; & Probability\;\left(P\right)=1-e^{-rt}\\ \text{Where}\\ r & =\text{selected rate}\\ t & =\text{ cycle length }\left(1\text{ year}\right) \end{array} \end{array}$$

### Model validation

The model was developed in consultation with the Committee; model structure, inputs, and results were presented to and discussed with the Committee for clinical validation and interpretation.

The model was systematically checked by the health economist undertaking the analysis (DD); this included inputting null and extreme values and checking that results were plausible given inputs. The model was peer reviewed by a second experienced health economist from the NGC (DW); this included systematic checking of the model calculations.

### Estimation of cost-effectiveness

When there are more than 2 comparators, as in this analysis, options must be ranked in order of increasing cost then options ruled out by dominance or extended dominance before calculating ICERs excluding these options. An option is said to be dominated, and ruled out, if another intervention is less costly and more effective. An option is said to be extendedly dominated if a combination of 2 other options would prove to be less costly and more effective.

It is also possible, for a particular cost-effectiveness threshold, to re-express cost-effectiveness results in term of net monetary benefit (NMB). This is calculated by multiplying the total QALYs for a comparator by the threshold cost per QALY value (for example, £20,000) and then subtracting the total costs (formula below). The decision rule then applied is that the comparator with the highest NMB, and hence the highest incremental NMB (INMB) vs. the model comparator, is the most cost-effective option at the specified threshold. That is the option that provides the highest number of QALYs at an acceptable cost.

$$\begin{array}{l} Net\;Monetary\;Benefit\;\left(X\right)=\left(QALYs\;\left(X\right)\times \lambda \right)-Costs\left(X\right)\\ Where:\lambda =threshold\;\left(£20,000\;per\;QALY\;gained\right)\\ \text{Cost}-\text{effective if}:\text{Highest net benefit} \end{array}$$

Results are also presented graphically where the incremental costs and incremental QALYs for each strategy compared to LMWH (std,std)+AES are shown on the cost-effectiveness plane. Scatter plots are also presented. We have also assessed the decision uncertainty by calculating the probability of being the most cost-effective option at the specified cost-effectiveness threshold.

## Results

### Base case- eTHR

**Figure 2:**
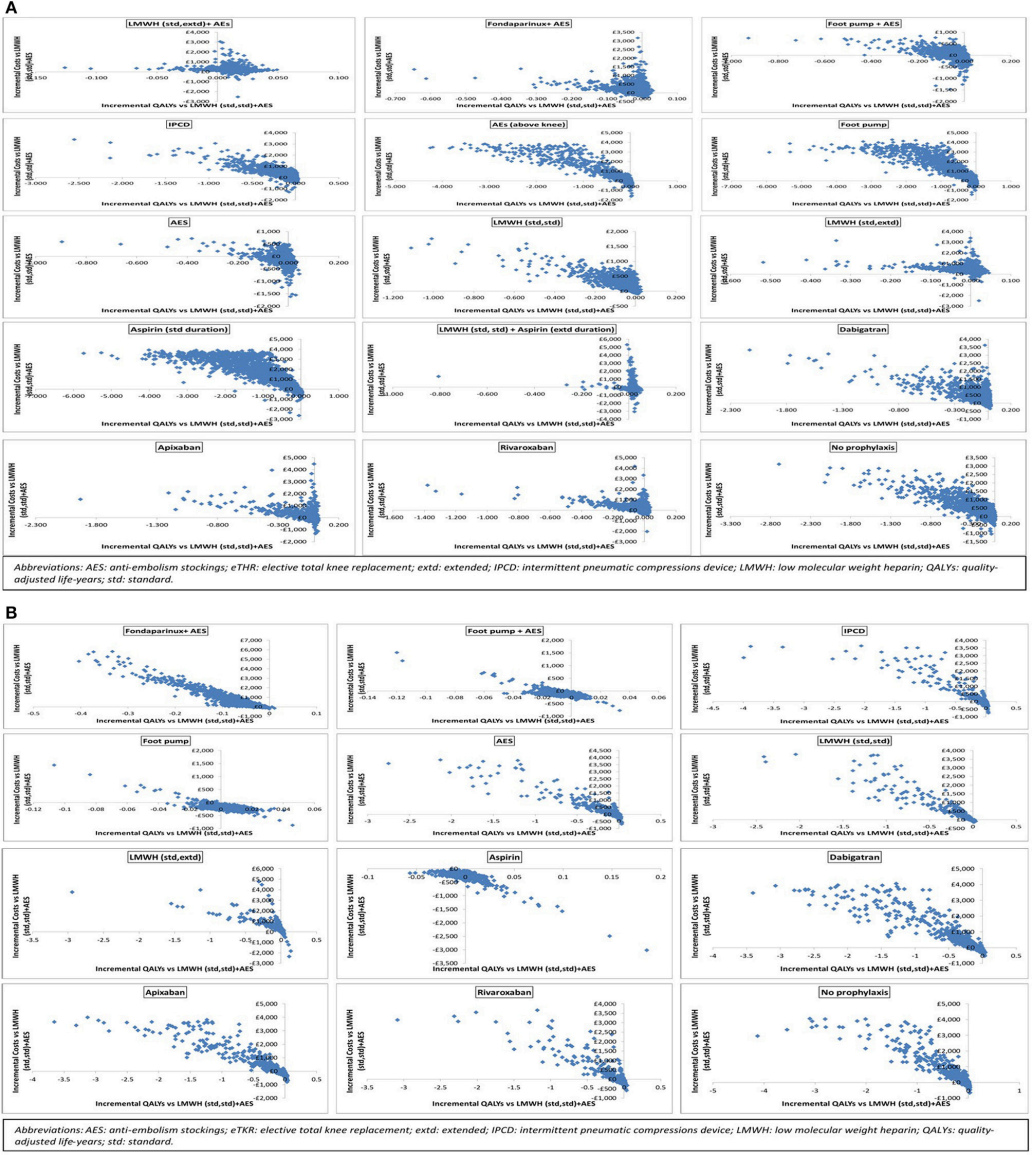
Scatter plots showing the probabilistic analysis results for **(A)** elective total hip replacement (eTHR) and **(B)** elective total knee replacement (eTKR) populations.

**Figure 3:**
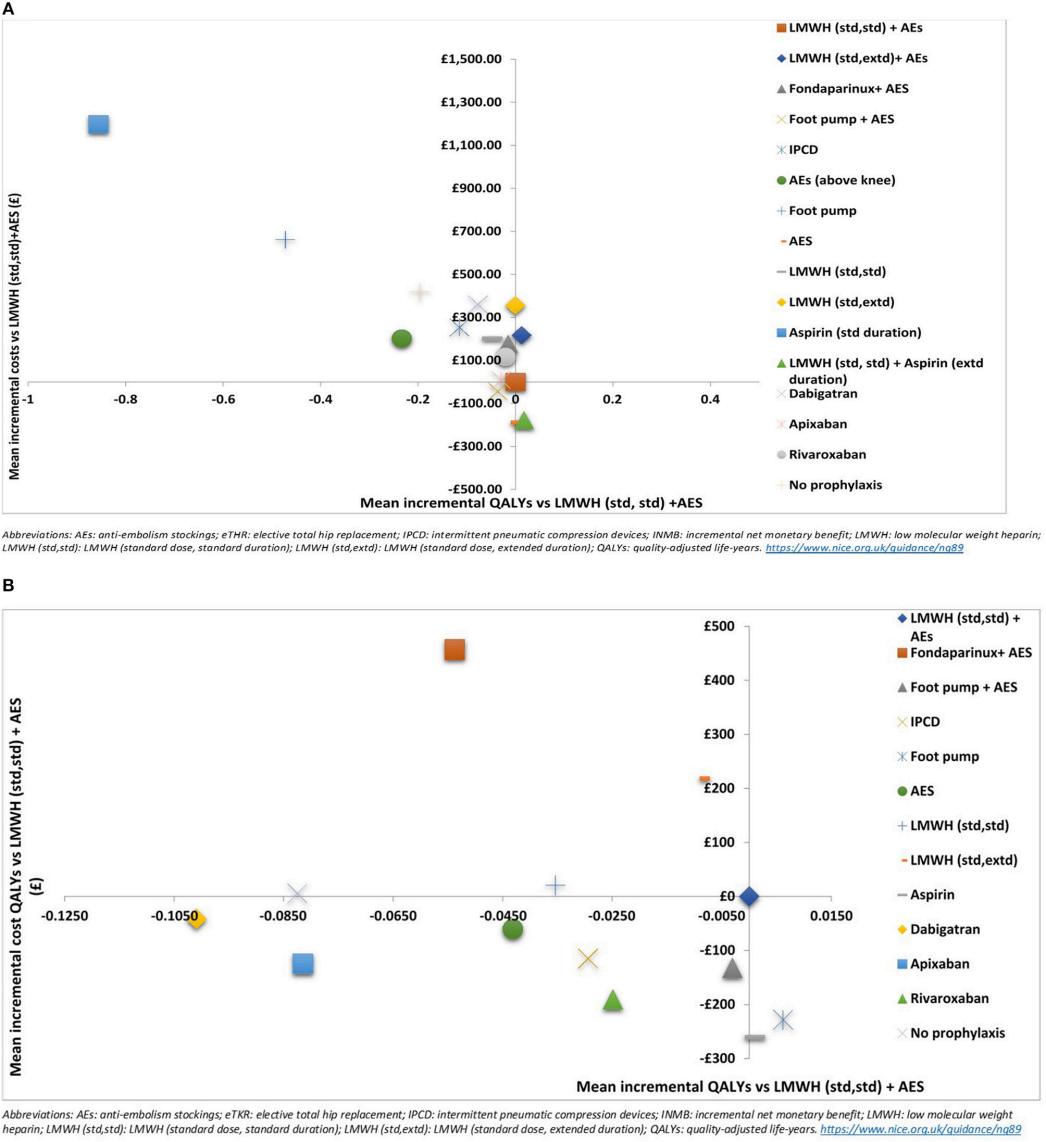
Cost effectiveness plane showing the mean incremental costs and QALYs compared to LMWH (standard dose, standard duration) + AES for **(A)** elective total hip replacement (eTHR) and **(B)** elective total knee replacement (eTKR) populations.

The results of the probabilistic base case analysis for the eTHR population are presented in Table 7 and on the cost-effectiveness plane as scatter plots in Figure 2A and as point estimates in Figure 3A. These show that the most effective option, with the highest mean QALYs-gained over lifetime per person, was the prophylaxis strategy consisting of LMWH (standard dose, standard duration) followed by aspirin (low dose, extended duration; 10.293 discounted QALYs; 95% CI: 8.02 to 12.00). It was followed closely by LMWH (standard dose, extended duration) + AES (10.288; 95% CI: 8.02 to 12.00). The highest cost option was aspirin (low dose, standard duration), with mean discounted cost of £1,687 (95% CI: £157 to £4,039) per person. The lowest cost prophylaxis strategy was AES, with mean discounted cost per person of £299 (95% CI: £102 to £793) followed by the strategy of LMWH (standard dose, standard duration) followed by aspirin (low dose, extended duration) which had a mean discounted cost of £311 (95% CI: £148 to £1437).

**Table 7:** Base case probabilistic analysis results for A) Elective total hip replacement B) Elective total knee replacement.

**Intervention**	**Mean discounted QALYs (95% CI)**	**Mean Discounted Costs (95% CI)**	**Incremental QALYs vs. LMWH+ AES (95% CI)**	**Incremental costs vs. LMWH+ AES (95% CI)**	**Mean INMB at £20K per QALY (95% CI)**	**Probability most CE at £20k (a)**	**Rank at £20k (95% CI) (b)**
**Elective total hip replacement (eTHR)**							
LMWH (std,std) + AES (c)	10.28 (8.01 to 11.98)	£489 (£350 to £832)	0.000 (0.000 to 0.000)	£0 (£0 to £0)	£0 (£0 to £0)	0.1%	4 (3, 11)
LMWH (std,extd)+ AES	10.29 (8.02 to 12.00)	£706 (£509 to £1,376)	0.013 (−0.004 to 0.030)	£217 (–£42 to £694)	£36 (–£745 to £484)	0.6%	2 (2, 12)
Fondaparinux+ AES	10.26 (7.98 to 11.96)	£665 (£336 to £1,563)	−0.015 (−0.112 to 0.013)	£176 (–£92 to £800)	–£478 (–£2,618 to £278)	0.2%	6 (3, 15)
Foot pump + AES	10.24 (7.99 to 11.94)	£445 (£209 to £926)	−0.036 (−0.182 to 0.012)	–£44 (–£329 to £398)	–£684 (–£3,930 to £478)	0.6%	9 (2, 15)
IPCD	10.16 (7.86 to 11.91)	£742 (£255 to £1,968)	−0.115 (−0.681 to 0.011)	£253 (–£246 to £1,455)	–£2,550 (–£14,733 to £396)	0.1%	12 (4, 15)
AES (above knee)	10.04 (7.35 to 11.93)	£691 (£119 to £3,765)	−0.234 (−2.197 to 0.027)	£202 (–£424 to £3,310)	–£4,873 (–£46,725 to £861)	13.2%	14 (1, 16)
Foot pump	9.80 (6.96 to 11.77)	£1,150 (£161 to £4,054)	−0.472 (−2.681 to 0.015)	£661 (–£344 to £3,578)	–£10,104 (–£57,043 to £590)	1.4%	15 (2, 16)
AES	10.27 (8.01 to 11.97)	£299 (£102 to £793)	−0.009 (−0.103 to 0.022)	–£189 (–£460 to £261)	£5 (–£2,106 to £781)	8.4%	3 (1, 14)
LMWH (std,std)	10.23 (7.95 to 11.94)	£691 (£375 to £1,413)	−0.048 (−0.283 to 0.009)	£202 (–£44 to £767)	–£1,162 (–£6,266 to £197)	0.0%	10 (6, 13)
LMWH (std,extd)	10.27 (7.98 to 11.98)	£844 (£528 to £1,582)	0.000 (−0.070 to 0.025)	£356 (£24 to £954)	–£361 (–£2,042 to £349)	0.1%	5 (4, 13)
Aspirin (low dose, std duration)	9.42 (6.50 to 11.59)	£1,687 (£157 to £4,039)	−0.856 (−3.179 to 0.009)	£1,198 (–£390 to £3,610)	–£18,312 (–£66,988 to £479)	0.7%	16 (2, 16)
LMWH (std, std) + Aspirin (extd duration)	10.29 (8.02 to 12.00)	£311 (£148 to £1437)	0.018 (0.003 to 0.036)	–£178 (–£548 to £781)	£530 (–£784 to £1,103)	72.0%	1 (1, 11)
Dabigatran	10.20 (7.93 to 11.94)	£849 (£319 to £1,957)	−0.077 (−0.465 to 0.010)	£360 (–£122 to £1,331)	–£1,903 (–£10,144 to £254)	0.0%	11 (5, 15)
Apixaban	10.25 (7.96 to 11.97)	£497 (£163 to £1,588)	−0.030 (−0.270 to 0.022)	£8 (–£302 to £895)	–£598 (–£6,089 to £632)	2.2%	8 (2, 14)
Rivaroxaban	10.25 (7.97 to 11.97)	£606 (£227 to £1,452)	−0.021 (−0.190 to 0.019)	£117 (–£234 to £814)	–£529 (–£4,385 to £514)	0.4%	7 (2, 13)
No prophylaxis	10.08 (7.80 to 11.82)	£908 (£297 to £2,185)	−0.196 (−0.885 to −0.008)	£419 (–£195 to £1,677)	–£4,336 (–£19,297 to –£95)	0.0%	13 (10, 16)
**Elective total knee replacement (eTKR)**							
LMWH (std,std) + AES (c)	9.81 (7.86 to 11.58)	£448 (£364 to £613)	0.000 (0.000 to 0.000)	£0 (£0 to £0)	£0 (£0 to £0)	0.1%	4 (4, 12)
Fondaparinux+ AES	9.75 (7.83 to 11.52)	£904 (£358 to £3016)	−0.054 (−0.183 to −0.009)	£457 (–£53 to £2466)	–£1,532 (–£6,183 to –£176)	0.0%	11 (6, 13)
Foot pump + AES	9.80 (7.86 to 11.58)	£315 (£208 to £590)	−0.003 (−0.020 to 0.006)	–£132 (–£234 to £32)	£72 (–£379 to £343)	0.1%	3 (3, 12)
IPCD	9.78 (7.82 to 11.56)	£332 (£133 to £1246)	−0.029 (−0.367 to 0.019)	–£115 (–£304 to £698)	–£473 (–£8,223 to £635)	5.8%	7 (1, 13)
Foot pump	9.81 (7.86 to 11.58)	£219 (£119 to £473)	0.006 (−0.011 to 0.018)	–£228 (–£332 to –£65)	£353 (–£101 to £665)	18.1%	1 (1, 10)
AES	9.76 (7.77 to 11.57)	£387 (£167 to £1397)	−0.043 (−0.420 to 0.014)	–£60 (–£271 to £876)	–£803 (–£9,251 to £520)	0.2%	9 (3, 13)
LMWH (std,std)	9.77 (7.79 to 11.55)	£468 (£287 to £1563)	−0.035 (−0.441 to 0.018)	£21 (–£105 to £989)	–£728 (–£10,057 to £445)	0.0%	8 (4, 11)
LMWH (std,extd)	9.80 (7.85 to 11.58)	£666 (£508 to £1302)	−0.009 (−0.111 to 0.023)	£218 (£34 to £832)	–£398 (–£3,013 to £397)	0.1%	6 (3, 12)
Aspirin (low dose, std duration)	9.81 (7.86 to 11.58)	£187 (£118 to £304)	0.001 (−0.018 to 0.014)	–£260 (–£436 to –£125)	£281 (–£195 to £703)	9.0%	2 (1, 12)
Dabigatran	9.71 (7.53 to 11.56)	£406 (£100 to £2987)	−0.101 (−1.308 to 0.020)	–£42 (–£343 to £2524)	–£1,977 (–£28,720 to £707)	3.6%	13 (1, 13)
Apixaban	9.73 (7.62 to 11.54)	£322 (£69 to £2624)	−0.081 (−1.178 to 0.023)	–£125 (–£392 to £2166)	–£1,504 (–£25,838 to £802)	42.8%	10 (1, 13)
Rivaroxaban	9.78 (7.79 to 11.57)	£256 (£82 to £1205)	−0.025 (−0.333 to 0.021)	–£191 (–£360 to £634)	–£306 (–£6,975 to £747)	19.7%	5 (1, 11)
No prophylaxis	9.73 (7.68 to 11.53)	£453 (£137 to £2281)	−0.082 (−0.894 to 0.014)	£6 (–£298 to £1,715)	–£1,655 (–£20,058 to £540)	0.4%	12 (3, 13)

*^a^ AES, anti-embolism stockings; CE, cost effective; CI, confidence interval; extd, extended; IPCD, intermittent pneumatic compression devices; INMB, incremental net monetary benefit; LMWH, low molecular weight heparin; QALYs, quality-adjusted life-years; std, standard*.

*Calculated at cost effectiveness threshold of £20,000 per QALY gained. ^b^ The rank is calculated based on the INMB. The intervention with the highest INMB is ranked first, The 95% CI has been calculated probabilistically. ^C^ Model comparator*.

Based on these results, the most cost-effective prophylaxis strategy for eTHR was LMWH (standard dose, standard duration) followed by aspirin (low dose, extended duration) with mean INMB vs. LMWH (standard dose, standard duration) +AES of £530 (95% CI: -£784 to £1,103). It also had the highest probability of being the most cost-effective option (72%). Other interventions which have a positive mean INMB compared with LMWH (standard dose, standard duration) +AES were: LMWH (standard dose, extended duration) + AES (INMB = £36; 95% CI: -£745 to £484) and AES (INMB = £5; 95% CI: -£2,106 to £781). However, compared to no prophylaxis, all interventions except three (aspirin [low dose, standard duration], foot pump and AES [above knee]) have positive INMB.

Among the mechanical prophylaxis interventions; AES seemed to be more cost-effective compared to IPCD and foot pumps, ranking 3rd (95% CI: 1 to 14) when length was unspecified. However, above-knee AES had negative INMB compared to no prophylaxis and ranked in the 14th place (see Table 7).

The DOACs (rivaroxaban, apixaban, and dabigatran) were dominant compared to no prophylaxis but were dominated by the model comparator (LMWH [standard dose, standard duration] +AES). Of the three DOACs, rivaroxaban was cost-effective compared to apixaban with an ICER of £12,242 per QALY-gained. Both rivaroxaban and apixaban were dominant (more effective and less costly) compared to dabigatran. The probability of being the most cost-effective was higher for apixaban (2.24%) compared to rivaroxaban (0.2%). However; there was more uncertainty around the ranking of apixaban, with a probability of being the least cost effective of 0.16% for apixaban compared to 0.08% for rivaroxaban.

The disaggregated health outcomes and costs are presented in Tables 8, 9. These show that the strategies that resulted in the lowest number of VTE events are LMWH (standard dose, standard duration) followed by aspirin (low dose, extended duration) and LMWH (standard dose, extended duration) + AES (8 [95%: 0 to 55] and 34 [95% CI: 5 to 116] per 1,000 persons; respectively). The highest number of VTE events was, not surprisingly, seen with the no prophylaxis strategy (491 per 1,000 (95% CI: 146 to 953). The number of surgical site bleeding events was highest for fondaparinux+ AES (51 per 1,000 [95% CI: 8 to 187]) followed by dabigatran with 44 per 1,000 [95% CI: 6 to 160]. Aspirin (low dose, standard duration) was associated with the highest number of PE, PTS, and CTEPH events (373, 60, and 11 per 1,000, respectively).

**Table 8:** Health outcomes per 1,000 for each prophylaxis strategy–eTHR population.

	**Intervention Short-term health outcomes (n [95% CI])**							**Long-term health outcomes (n [95% CI])**	
	**Symptomatic DVTs**	**Sympt proximal DVT**	**Asymptomatic DVTs**	**PEs**	**Total VTEs**	**Surgical site bleeding**	**Total deaths**	**PTS**	**CTEPH**
LMWH (std,std) + AES	9 (8 to 11)	8 (6 to 9)	46 (44 to 48)	7 (6 to 7)	62 (61 to 64)	28 (7 to 83)	1 (1 to 3)	7 (6 to 8)	0 (0 to 0)
LMWH (std,extd)+ AES	6 (1 to 19)	5 (1 to 16)	27 (4 to 96)	1 (0 to 9)	34 (5 to 116)	29 (2 to 131)	0 (0 to 2)	4 (1 to 13)	0 (0 to 0)
Fondaparinux+ AES	20 (7 to 42)	17 (6 to 35)	98 (36 to 204)	12 (1 to 52)	130 (52 to 263)	51 (8 to 187)	2 (0 to 11)	14 (6 to 30)	0 (0 to 2)
Foot pump + AES	25 (3 to 81)	21 (3 to 68)	122 (16 to 388)	22 (3 to 87)	169 (35 to 486)	13 (2 to 49)	5 (0 to 19)	19 (4 to 54)	1 (0 to 3)
IPCD	56 (10 to 134)	47 (8 to 111)	275 (49 to 634)	53 (2 to 299)	383 (79 to 858)	13 (2 to 49)	11 (0 to 62)	43 (9 to 99)	b (0 to 9)
AES (above knee)	16 (2 to 58)	14 (1 to 48)	80 (8 to 278)	106 (0 to 909)	203 (16 to 996)	13 (2 to 49)	23 (0 to 202)	26 (2 to 138)	3 (0 to 26)
Foot pump	17 (1 to 73)	14 (1 to 61)	84 (5 to 363)	213 (1 to 980)	314 (20 to 1078)	13 (2 to 49)	44 (0 to 243)	41 (2 to 152)	6 (0 to 30)
AES	20 (1 to 91)	16 (1 to 76)	97 (4 to 440)	11 (1 to 49)	127 (11 to 539)	13 (2 to 49)	2 (0 to 11)	14 (1 to 58)	0 (0 to 2)
LMWH (std,std)	34 (6 to 93)	28 (5 to 78)	168 (29 to 451)	25 (2 to 128)	227 (48 to 573)	28 (7 to 83)	5 (0 to 27)	26 (6 to 65)	1 (0 to 4)
LMWH (std,extd)	32 (3 to 100)	27 (3 to 83)	158 (17 to 482)	4 (0 to 32)	194 (22 to 589)	29 (2 to 131)	1 (0 to 6)	21 (2 to 65)	0 (0 to 1)
Aspirin (std duration)	10 (2 to 32)	8 (1 to 26)	49 (8 to 156)	373 (3 to 995)	433 (34 to 1066)	10 (8 to 12)	79 (1 to 288)	60 (4 to 155)	11 (0 to 31)
LMWH (std, std) + Aspirin	1 (0 to 8)	1 (0 to 7)	6 (0 to 42)	1 (0 to 6)	8 (0 to 55)	22 (0 to 190)	0 (0 to 1)	1 (0 to 6)	0 (0 to 0)
Dabigatran	48 (4 to 136)	40 (4 to 113)	233 (21 to 649)	37 (1 to 204)	317 (42 to 830)	44 (6 to 160)	8 (0 to 43)	36 (5 to 93)	1 (0 to 6)
Apixaban	7 (0 to 30)	6 (0 to 26)	33 (2 to 145)	21 (0 to 131)	61 (6 to 252)	42 (4 to 173)	4 (0 to 28)	7 (1 to 32)	1 (0 to 4)
Rivaroxaban	35 (4 to 110)	29 (3 to 92)	171 (19 to 527)	13 (0 to 88)	219 (28 to 651)	36 (4 to 138)	3 (0 to 18)	24 (3 to 73)	0 (0 to 3)
No prophylaxis	68 (16 to 139)	57 (13 to 115)	335 (80 to 669)	88 (8 to 384)	491 (146 to 953)	13 (2 to 49)	18 (1 to 82)	56 (16 to 112)	3 (0 to 12)

*^a^ AES, anti-embolism stockings; CTEPH, chronic thromboembolic pulmonary hypertension; DVT, deep vein thrombosis; eTHR, elective total hip replacement; extd, extended; IPCD, intermittent pneumatic compression devices; LMWH, low molecular weight heparin; PE, pulmonary embolism; PTS, post thrombotic syndrome; std, standard; VTE, venous thromboembolism*.

**Table 9:** Cost breakdown for each prophylaxis strategy per person–eTHR population.

**Intervention**	**Prophylaxis costs**	**VTE costs (95% CI)**	**All Bleeding costs (95% CI)**	**CTEPH costs (95% CI)**	**PTS costs (95% CI)**	**Post-amputation costs (95% CI)**	**Total costs (a) (95% CI)**
LMWH (std,std) + AES	£169	£11 (£10 to £11)	£210 (£72.8 to £554)	£19 (£15.4 to £23)	£60 (£52 to £69)	£20 (£13 to £27)	£489 (£350 to £833)
LMWH (std,extd)+ AES	£419	£4 (£5.1 to £13)	£217 (£39 to £847)	£4.2(£3 to £26)	£32 (£5 to £107)	£28 (£18 to £39)	£706 (£509 to £1,376)
Fondaparinux+ AES	£115	£20 (£5.8 to £59)	£375 (£92 to £1,248)	£32 (£2 to £144.5)	£124 (£49 to £254)	£0.00 (£0.00 to £0.00)	£665 (£336 to £1,563)
Foot pump + AES	£91	£32 (£7.3 to £103)	£99 (£23 to £334)	£60 (£7 to £228)	£163 (£34 to £456)	£0.00 (£0.00 to £0.00)	£445 (£209 to £926)
IPCD	£68	£75 (£11.3 to £327)	£99 (£23 to £334)	£129 (£4 to £654.5)	£371 (£78 to £847)	£0.00 (£0.00 to £0.00)	£742 (£255 to £1,968)
AES (above knee)	£50	£112 (£1.6 to £908)	£99 (£23 to £334)	£211 (£36 to £1,502)	£219 (£15 to £1,183)	£0.00 (£0.00 to £0.00)	£691 (£119 to £3,765)
Foot pump	£60	£218 (£4.7 to £978)	£99 (£23 to £334)	£420 (£3.5 to £1,632)	£354 (£19 to £1,300)	£0.00 (£0.00 to £0.00)	£1,150 (£161 to £4,054)
AES	£31	£19 (£2.5 to £61.7)	£99 (£23 to £334)	£30 (£2 to £136)	£121 (£11 to £498)	£0.00 (£0.00 to £0.00)	£299 (£102 to £793)
LMWH (std,std)	£138	£39 (£7.6 to £140)	£210 (£72.8 to £554)	£66 (£5 to £311)	£218 (£47 to £555)	£20 (£13 to £27)	£691 (£375 to £1,413)
LMWH (std,extd)	£387	£17 (£2.4 to £54.7)	£217 (£39 to £847)	£12 (£0.1 to £87)	£181 (£21 to £551)	£28 (£18 to £39)	£845 (£528 to £1,582)
Aspirin (std duration)	£0.24	£374 (£7.2 to £989)	£98 (£82 to £119)	£702 (£8 to £1,687)	£512 (£34 to £1,322)	£000 (£000 to £000)	£1,687 (£157 to £4,034)
LMWH (std, std) + Aspirin	£115	£1.4 (£2 to £9)	£163 (£11 to £1,225)	£3 (£0 to £18)	£7.5 (£0.01 to £54)	£20 (£13 to £27)	£311 (£148 to £1,437)
Dabigatran	£80	£55.6 (£7.5 to £227)	£316 (£75.5 to £1,048)	£93 (£4 to £487)	£305 (£42 to £795)	£0.00 (£0.00 to £0.00)	£849 (£319 to £1,957)
Apixaban	£59	£23.5 (£1.5 to £132.6)	£298 (£56.5 to £1,139)	£53 (£1 to £321)	£63 (£6.5 to £270)	£0.00 (£0.00 to £0.00)	£497 (£163 to £1,588)
Rivaroxaban	£74	£27 (£3.4 to £105)	£265 (£58.6 to £907)	£34 (£0.4 to £225)	£206 (£28 to £629)	£0.00 (£0.00 to £0.00)	£606 (£227 to £1,452)
No prophylaxis	£0	£115 (£26 to £416)	£99 (£23 to £334)	£213 (£24 to £810)	£481 (£140 to £957)	£0.00 (£0.00 to £0.00)	£908 (£297 to £2,185)

*^a^ AES, anti-embolism stockings; CI, confidence interval; eTHR, elective total hip replacement; extd, extended; IPCD, intermittent pneumatic compression devices; LMWH, low molecular weight heparin; std, standard; VTE, venous thromboembolism; CTEPH, chronic thromboembolic pulmonary hypertension; PTS, post thrombotic syndrome. (a) May not exactly equal the sum of the components due to rounding*.

The breakdown of costs for all prophylaxis strategies is in line with the results for health outcomes. The cost of the prophylaxis itself was highest for LMWH (standard duration, extended duration)+ AES (£419 per person); driven by the high administration and monitoring costs for an extended duration.

### Base case- eTKR

The results of the probabilistic base case analysis for the eTKR population are presented in Table 7. Figure 2B presents the cost-effectiveness plane with scatter plots while the point estimates are presented in Figure 3B. These showed that the most effective option, with the highest mean gain in QALYs over lifetime per person, was foot pump (9.814 [95% CI: 7.86 to 11.58] discounted QALYs gained). This was followed closely by aspirin (low dose, standard duration) with a mean of 9.809 (95% CI: 7.86 to 11.58) and LMWH (standard dose, standard duration) + AES with a mean of 9.807 (95% CI: 7.86 to 11.58). The highest cost option was fondaparinux + AES, with mean discounted costs £904 (95% CI: £358 to £3,016). The lowest cost prophylaxis strategy was aspirin (low dose, standard duration), with mean discounted costs of £187 (95% CI: £118 to £304).

Based on these results, the most cost-effective prophylaxis strategy, with the highest NMB, was foot pump with mean INMB vs. LMWH (standard dose, standard duration) + AES of £353 (95% CI: -£101 to £665) followed by aspirin (low dose, standard duration) with mean INMB of £281 (95% CI: -£195 to £703). However, the results show considerable uncertainty where the most cost-effective option (foot pump) rank having a 95% CI of 1 to 10 around the mean rank and a probability of being the most cost-effective of only 18%. The only interventions with positive INMB when compared with LMWH (std, std) + AES were foot pump, aspirin, and the combined strategy of foot pump + AES. Compared to no prophylaxis, though, all interventions had a positive INMB except dabigatran.

Of the DOACs included in the model; rivaroxaban dominated both apixaban and dabigatran. However, the model comparator (LMWH [standard dose, standard duration] + AES) was cost effective compared to rivaroxaban (ICER: £7,686). The probability of being the most cost-effective was higher for apixaban (44%) compared to rivaroxaban (18%). However; there was more uncertainty around the ranking of apixaban, with a 5% probability of it being the least cost effective compared to 0% for rivaroxaban.

The disaggregated health outcomes and costs for all prophylaxis strategies are presented in Tables 10, 11 These show that rivaroxaban had the lowest number of VTE events (60 per 1,000 persons [95% CI: 14 to 211]). The number of surgical site bleeding events was highest for fondaparinux + AES (79 per 1,000 [95% CI: 2 to 411]) followed by rivaroxaban (16 per 1000 [95% CI: 1 to 67]). The “no prophylaxis” strategy was associated with the highest number of PTS events (23 per 1,000 [7 to 81]), Dabigatran had the highest number of PE events (51 per 1,000 [0 to 644]). The disaggregate costs were in line with the results for

**Table 10:** Health outcomes breakdown per 1,000 for each prophylaxis strategy - eTKR population.

**Intervention**	**Short-term health outcomes [n (95% CI)]**							**Long-term health outcomes [n(95% CI)]**	
	**Symptomatic DVT**	**Sympt proximal DVT**	**Asymptomatic DVT**	**PE**	**Total VTE**	**Surgical site bleeding**	**Total deaths**	**PTS**	**CTEPH**
LMWH (std,std) + AES	6 (5 to 8)	1 (0 to 2)	134 (132 to 136)	4 (4 to 5)	144 (143 to 146)	9 (1 to 32)	1 (0 to 2)	8 (6 to 11)	0 (0 to 0)
Fondaparinux+ AES	6 (2 to 13)	1 (0 to 3)	121 (36 to 261)	10 (2 to 25)	136 (46 to 284)	79 (2 to 411)	2 (0 to 6)	8 (3 to 16)	0 (0 to 1)
Foot pump + AES	9 (4 to 15)	2 (0 to 4)	181 (91 to 311)	6 (3 to 11)	195 (101 to 333)	12 (1 to 51)	1 (0 to 3)	10 (5 to 19)	0 (0 to 0)
IPCD	10 (3 to 19)	2 (0 to 5)	202 (66 to 405)	19 (0 to 175)	230 (71 to 495)	12 (1 to 51)	4 (0 to 35)	13 (4 to 38)	1 (0 to 5)
Foot pump	4 (0 to 12)	1 (0 to 3)	79 (11 to 243)	3 (0 to 9)	85 (14 to 259)	12 (1 to 51)	1 (0 to 2)	5 (1 to 14)	0 (0 to 0)
AES	13 (6 to 22)	3 (1 to 6)	285 (144 to 465)	24 (0 to 203)	323 (158 to 567)	12 (1 to 51)	5 (0 to 39)	18 (8 to 48)	1 (0 to 6)
LMWH (std,std)	4 (1 to 9)	1 (0 to 2)	89 (30 to 195)	21 (0 to 232)	114 (33 to 337)	9 (1 to 32)	4 (0 to 44)	8 (2 to 37)	1 (0 to 7)
LMWH (std,extd)	4 (1 to 10)	1 (0 to 2)	76 (18 to 204)	8 (0 to 49)	88 (19 to 238)	10 (0 to 68)	2 (0 to 10)	5 (1 to 16)	0 (0 to 1)
Aspirin	7 (2 to 17)	1 (0 to 4)	149 (39 to 367)	5 (1 to 12)	160 (45 to 390)	9 (8 to 11)	1 (0 to 3)	9 (2 to 20)	0 (0 to 0)
Dabigatran	4 (1 to 10)	1 (0 to 2)	88 (27 to 199)	51 (0 to 644)	142 (32 to 722)	11 (1 to 45)	11 (0 to 127)	12 (2 to 98)	2 (0 to 19)
Apixaban	2 (1 to 6)	0 (0 to 1)	51 (15 to 121)	44 (0 to 568)	97 (18 to 606)	8 (0 to 35)	9 (0 to 102)	9 (1 to 85)	1 (0 to 16)
Rivaroxaban	2 (1 to 5)	0 (0 to 1)	42 (11 to 104)	16 (0 to 163)	60 (14 to 211)	16 (1 to 67)	3 (0 to 34)	4 (1 to 24)	0 (0 to 5)
No prophylaxis	15 (6 to 27)	3 (1 to 7)	328 (132 to 565)	41 (0 to 429)	385 (151 to 781)	12 (1 to 51)	8 (0 to 87)	23 (7 to 81)	1 (0 to 13)

*AES, anti-embolism stockings; CTEPH, chronic thromboembolic pulmonary hypertension; DVT, deep vein thrombosis; eTKR, elective total knee replacement; extd, extended; IPCD, intermittent pneumatic compression devices; LMWH, low molecular weight heparin; PE, pulmonary embolism; PTS, post thrombotic syndrome; std, standard; VTE, venous thromboembolism*.

**Table 11:** Cost breakdown for each prophylaxis strategy per person–eTKR population.

**Intervention**	**Prophylaxis costs**	**VTE costs (95% CI)**	**All Bleeding costs (95% CI)**	**CTEPH costs (95% CI)**	**PTS costs (95% CI)**	**Post-amputation costs (95% CI)**	**Total costs (a) (95% CI)**
LMWH (std,std) + AES	£142	£6 (£5 to £6)	£93 (£32 to £260)	£13 (£10 to £15)	£67 (£52 to £99)	£101 (£69 to £142)	£448 (£364 to £613)
Fondaparinux+ AES	£128	£11 (£3 to £26)	£671 (£140 to £2,769)	£27 (£7 to £72)	£67 (£25 to £139)	£0.00 (£0.00 to £0.00)	£904 (£358 to £3,016)
Foot pump + AES	£91	£8 (£4 to £13)	£109 (£30 to £371)	£17 (£8 to £33)	£91 (£46 to £165)	£0.00 (£0.00 to £0.00)	£315 (£208 to £590)
IPCD	£42	£21 (£0.9 to £177)	£109 (£30 to £371)	£45 (£0.001 to £448)	£116 (£31 to £337)	£0.00 (£0.00 to £0.00)	£333 (£133 to £1,246)
Foot pump	£60	£4 (£0.8 to £10)	£109 (£30 to £371)	£8 (£1.0 to £25)	£40 (£7 to £118)	£0.00 (£0.00 to £0.00)	£219 (£119 to £473)
AES	£31	£27 (£2 to £203)	£109 (£30 to £371)	£59 (£0.2 to £485)	£161 (£66 to £401)	£0.00 (£0.00 to £0.00)	£387 (£167 to £1,397)
LMWH (std,std)	£111	£21 (£0.4 to £231)	£93 (£32 to £260)	£49 (£0.001 to £572)	£67 (£14.5 to £328)	£101 (£69 to £142)	£468 (£287 to £1,563)
LMWH (std,extd)	£356	£9 (£0.2 to £50)	£107 (£21 to £511)	£19 (£0.00 to £130)	£46 (£8 to £137)	£103 (£68 to £150)	£666 (£508 to £1,302)
Aspirin	£0.49	£6 (£2 to £14)	£92 (£70 to £130)	£14 (£3 to £36)	£74 (£21 to £178)	£0.00 (£0.00 to £0.00)	£187 (£118 to £304)
Dabigatran	£34	£51 (£0.4 to £640)	£106 (£32 to £34)	£111 (£0.002 to £1,322)	£104 (£14 to £867)	£0.00 (£0.00 to £0.00)	£406 (£100 to £2,987)
Apixaban	£23	£44 (£0.2 to £564)	£80 (£23 to £254)	£97 (£0.002 to £1,157)	£79 (£8 to £753)	£0.00 (£0.00 to £0.00)	£322 (£69 to £2,624)
Rivaroxaban	£25	£16 (£0.16 to £162)	£139 (£38 to £470)	£37 (£0.00 to £388)	£39 (£6 to £214)	£0.00 (£0.00 to £0.00)	£256 (£82 to £1,206)
No prophylaxis	£0	£44 (£2 to £429)	£109 (£30 to £371)	£97 (£0.05 to £962)	£203 (£64 to £701)	£0.00 (£0.00 to £0.00)	£453 (£137 to £2,281)

*AES, anti-embolism stockings; eTKR, elective total hip replacement; extd, extended; IPCD, intermittent pneumatic compression devices; LMWH, low molecular weight heparin; std, standard; VTE, venous thromboembolism; CTEPH, chronic thromboembolic pulmonary hypertension; PTS, post thrombotic syndrome. (a) May not exactly equal the sum of the components due to rounding*.

health outcomes. The cost of the prophylaxis itself was highest for LMWH (standard dose, extended duration) at £356 per person.

### Sensitivity analyses

For the eTHR population, the results of the SAs show that the most cost-effective option (LMWH [standard dose, standard duration] followed by aspirin [low dose, extended duration]) remained the same in all SAs except in SA4 where the mean age of the cohort was reduced to 40 years; where it dropped to the second rank and LMWH (standard dose, standard duration) + AES became the most cost effective.

For the eTKR population, the optimal strategy (foot pump) remained the same in all SAs. Dabigatran was the least cost effective in all SAs.

## Discussion

To our knowledge, this is the most comprehensive and up-to-date economic evaluation of VTE prophylaxis strategies to be conducted from the English NHS and PSS perspective. The results showed that, for eTHR, the most cost-effective prophylaxis strategy is LMWH (standard dose, standard duration) followed by aspirin (low dose, extended duration) with mean INMB £530 (95% CI: -£784 to £1,103). It also has the highest probability of being the most cost-effective option (72%). Where parenteral options are not acceptable or contraindicated; rivaroxaban would be the most cost-effective prophylaxis option. Of the mechanical prophylaxis options considered in the analysis; AES-based strategies appeared to be the more cost-effective compared to IPCDs and foot pumps.

For eTKR, foot pumps appeared to be the most cost-effective option with mean INMB of £353 (95% CI: -£101 to £665), though with a low (18%) probability of being the most cost-effective option. It was followed closely by aspirin (low dose, standard duration) with mean INMB of £281 (95% CI: -£195 to £703). However, the results of the analysis in the eTKR population were highly uncertain and should be interpreted with caution. Indeed, the guideline committee did not feel that recommending foot pump in this population would be appropriate if pharmacological prophylaxis is a suitable option, as it is likely to delay mobilization and, consequently, discharge. Instead, the committee recommended aspirin (low dose, standard duration) as first option. Foot pump/IPCD, however, was recommended for those who have contraindications to pharmacological prophylaxis.

The different results for the two populations can be explained in terms of the differences in the baseline risk inputs used, particularly the risk of symptomatic proximal DVT which also has the highest probability of developing PTS in the longer term. This risk was much higher in the eTHR population which made the use of more costly, longer duration prophylaxis options cost effective as the costs of these strategies are offset by the savings achieved from avoiding these costly events.

The relative cost effectiveness of the DOACs in this population was similar to that in the eTHR population, with rivaroxaban being the most cost effective of the three DOACs considered, though the uncertainty was clearly higher than that seen in the eTHR analysis, with very wide 95% CI around the ranking of these interventions.

To our knowledge, this analysis is the first to include all established interventions for primary prevention of hospital-acquired VTE in eTHR and eTKR that are relevant to clinical practice in the English NHS; including mechanical, pharmacological, and combination prophylaxis. It is also the first to account for some of the consequences of HIT, including amputation, and to completely account for the consequences of major bleeding including joint infections, wound haematoma and return to theater. These improvements represent important step forward in modeling VTE prophylaxis compared to previously published models.

The model structure represented both the acute phase in the immediate post-operative period as well as the long-term phase to life-time time horizon; using a Markov model to capture long-term consequences of VTE events including PTS and CTEPH. It has been based on NMAs of the three main outcomes DVT, PE, and MB. This is similar to the most recently published UK analysis, which conducted NMAs of these outcomes.

However, we have attempted to address the limitations of the previously published models, including the NICE guideline CG92 model, that were highlighted by the orthopedic surgery community. For example, the lack of differentiation between proximal and distal DVT was a major concern. We have addressed this issue by differentiating between proximal and distal DVT for both symptomatic and asymptomatic events, allowing for different probabilities of progression from each of these to clinical sequelae. We emphasized the fact that asymptomatic DVT also does not have an impact on costs and outcomes in the short term as it is not diagnosed in clinical practice.

There was also previous criticism of the baseline risk used in the model, which was based on data from the “no prophylaxis arm” in historical trials from the 1970s. We have used baseline risk estimates from observational cohort studies that used the NJR data (Jameson et al., 2011, 2012). This is in line with the NICE Reference Case specifications, which considers the most appropriate source of baseline risk is large observational cohort studies that describe the natural history of the disease/condition of interest in the target population.

Overall, published economic evaluations that compared VTE prophylaxis to no prophylaxis in eTHR and eTKR concluded that prophylaxis was a cost-effective intervention (National Colloborating Centre for Acute Care, 2007; National Clinical Guideline Centre, 2010). The choice of an optimum prophylaxis strategy, however, varied across studies and among countries. This is partly explained by the difference in the range of interventions included in each of these studies but also by the differences in acquisition costs and sources of effectiveness evidence. Our analysis is concordant with the conclusion reached by Brockbank and Wolowacz (2017) that the difference between the included interventions in terms of QALYs-gained is very small and the results are likely to be more sensitive to differences in costs.

The results also showed that out of the DOACs considered; rivaroxaban is likely to be the most cost-effective. This was seen particularly in the eTHR population where rivaroxaban dominated dabigatran and was cost-effective compared to apixaban with an ICER of £12,242 per QALY-gained. This finding was in line with the results of NICE TA170, where rivaroxaban was found to dominate dabigatran (National Institute for Health and Clinical Excellence, 2009).

A recent analysis funded by the NIHR found that rivaroxaban dominated dabigatran and was cost-effective compared to apixaban with an ICER of £114 per QALY gained (Sterne et al., 2016). TA245 also found that dabigatran was dominated, apixaban was extendedly dominated and rivaroxaban had an ICER of £22,123 per QALY-gained compared to fondaparinux (National Institute for Health and Clinical Excellence, 2012).

In eTKR, rivaroxaban dominated both apixaban and dabigatran. This was in line with the results of the economic models assessed as part of NICE TA170 and TA245 and a more recent analysis funded by the NIHR (National Institute for Health and Clinical Excellence, 2009, 2012; Sterne et al., 2016).

However; our analysis showed that LMWH in combination with AES, which is the most commonly prescribed prophylaxis strategy in the UK, is more cost effective than the DOACs. This is in accordance with Kapoor et al. (2010) who concluded that fondaparinux and extended duration LMWH can be cost-effective strategies. However, in our analysis, fondaparinux was not considered cost effective due to the high risk of bleeding and the costs associated with it.

Despite all our efforts, though, the results of this economic analysis for the eTKR population remain uncertain. This is largely due to the uncertainty and imprecision of the NMA results that informed it; which resulted from the sparse nature of the networks for the PE and MB outcomes in this population. This made it difficult to precisely estimate the relative effectiveness of the interventions. The resulting imprecise estimates of cost effectiveness preclude defining a clear ranking of the included interventions in terms of their cost-effectiveness. However, this is a reflection of the state of the collective body of evidence in this clinical area; which calls for future research to focus on producing better quality evidence that assesses the relative efficacy of VTE prophylaxis in eTKR.

The results of this analysis have also been largely based on epidemiological and cost data specific to England including the cohort characteristics which were based on data from the NJR. Additionally, the interventions included in the analysis were true to current clinical practice. Furthermore, this analysis has been undertaken from the NHS and PSS perspective. These factors may limit the generalisability to other populations and settings. However, the relative efficacy estimates were based on comprehensive SR and NMAs that did not restrict the inclusion of studies to specific countries.

Nevertheless, this economic evaluation is the most comprehensive and up-to-date assessment of the cost effectiveness of VTE prophylaxis strategies in major orthopedic surgeries. Its results informed the NICE guideline committee's recommendation which defined the VTE prophylaxis options to be used in eTHR and eTKR in the English NHS hospitals.

## Conclusions

To conclude, in people undergoing elective total hip and elective total knee replacement surgeries, VTE prophylaxis appears to be cost effective compared to no prophylaxis. A strategy consisting of LMWH for 10 days followed by aspirin for 28 days was the most cost-effective for elective total hip replacement. For elective total knee replacement, the results were highly uncertain. Foot pump appeared to be the most cost-effective strategy followed closely by aspirin (low dose). However, these results should be interpreted with caution within the context of current clinical practice and combined with other considerations including patient preferences to inform prescribing decisions. Future research should focus on assessing cost-effectiveness of VTE prophylaxis in the eTKR population.

## Data availability statements

Datasets are available on request by emailing the National Institute for Health and Care Excellence (NICE) nice@nice.org.uk and citing the guideline number NG89.

## Author contributions

DD designed the study, collected the data, undertook the analysis, contributed to the interpretation of results, and wrote the manuscript. DW contributed to the study design, quality assured the analysis, and contributed to the interpretation of the results. SL and JG conducted the NMAs and contributed to the data analysis and interpretation of the results. XG, MR, NR, BH, GS, JC, CS, and PB contributed to the study design, acquisition of data, and interpretation of results. All authors revised the manuscript critically and approved its submission.

### Conflict of interest statement

XG reports grants from NIHR Clinician Scientist (Open Fracture Management, grants from NIHR RfPB Programme (TULIP trial), grants from NIHR SRP (Hip fracture review portfolio), grants from NIHR RfPB Programme (WHiTE5 definitive trial), grants from NIHR HTA Programme (TRAFFix Trial), grants from NIHR RfPB Programme (WHiTE5 feasibility), grants from X-Bolt Commercial Grant (Investigator Initiated), outside the submitted work. MR reports personal fees from Zimmer, grants from Heraeus cement, grants from ConvaTec, grants and personal fees from ASHN/Heraeus, grants from The Health Foundation, grants and personal fees from Stryker, grants from Heraeus, grants from Orthopaedic Research UK, grants from Orthopaedic Research UK, grants from AR-UK, grants and personal fees from 3 m Healthcare, grants and personal fees from The Health Foundation, grants from OR-UK, grants from Curetis, grants and personal fees from Heraeus, grants and personal fees from NHSI, Vifor Pharma, Shuelke, Northumbria NHS Vangaurd, grants from Depuy, Stryker, Zimmer Biomet, Biocomposites, Aquilant, Heraeus, personal fees from Zimmer Bioment, personal fees from Heraeus, personal fees from Stryker, other from ConvaTec, outside the submitted work. PB reports personal fees from Royal College of Physicians, during the conduct of the study. The remaining authors declare that the research was conducted in the absence of any commercial or financial relationships that could be construed as a potential conflict of interest.

## Abbreviations

AES: anti-embolism stockings
CRNMB: clinically-relevant non-major bleeding
CTEPH: chronic thromboembolic pulmonary hypertension
CUA: cost utility analysis
DVT: deep vein thrombosis
eTHR: elective total hip replacement
eTKR: elective total knee replacement
HIT: Heparin-induced thrombocytopaenia
ICER: incremental cost effectiveness ratio
LMWH: low molecular weight heparin
MB: major bleeding
NICE: National Institute for Health and Care Excellence
NJR: National Joint Registry
NMA: network meta-analysis
PE: pulmonary embolism
PTS: post thrombotic syndrome
QALYs: quality adjusted life years
Std: standard
VTE: venous thromboembolism.
